# Electronic Consultation in Primary Care Between Providers and Patients: Systematic Review

**DOI:** 10.2196/13042

**Published:** 2019-12-03

**Authors:** Freda Mold, Jane Hendy, Yi-Ling Lai, Simon de Lusignan

**Affiliations:** 1 Faculty of Health and Medical Sciences University of Surrey Guildford United Kingdom; 2 Brunel Business School Brunel University London Uxbridge United Kingdom; 3 Faculty of Business and Law University of Portsmouth Portsmouth United Kingdom; 4 Nuffield Department of Primary Care Health Science University of Oxford Oxford United Kingdom

**Keywords:** referral and consultation, health services accessibility, primary health care, general practice, patient access to records, patient portals, Web-based access

## Abstract

**Background:**

Governments and health care providers are keen to find innovative ways to deliver care more efficiently. Interest in electronic consultation (e-consultation) has grown, but the evidence of benefit is uncertain.

**Objective:**

This study aimed to assess the evidence of delivering e-consultation using secure email and messaging or video links in primary care.

**Methods:**

A systematic review was conducted on the use and application of e-consultations in primary care. We searched 7 international databases (MEDLINE, EMBASE, CINAHL, Cochrane Library, PsycINFO, EconLit, and Web of Science; 1999-2017), identifying 52 relevant studies. Papers were screened against a detailed inclusion and exclusion criteria. Independent dual data extraction was conducted and assessed for quality. The resulting evidence was synthesized using thematic analysis.

**Results:**

This review included 57 studies from a range of countries, mainly the United States (n=30) and the United Kingdom (n=13). There were disparities in uptake and utilization toward more use by younger, employed adults. Patient responses to e-consultation were mixed. Patients reported satisfaction with services and improved self-care, communication, and engagement with clinicians. Evidence for the acceptability and ease of use was strong, especially for those with long-term conditions and patients located in remote regions. However, patients were concerned about the privacy and security of their data. For primary health care staff, e-consultation delivers challenges around time management, having the correct technological infrastructure, whether it offers a comparable standard of clinical quality, and whether it improves health outcomes.

**Conclusions:**

E-consultations may improve aspects of care delivery, but the small scale of many of the studies and low adoption rates leave unanswered questions about usage, quality, cost, and sustainability. We need to improve e-consultation implementation, demonstrate how e-consultations will not increase disparities in access, provide better reassurance to patients about privacy, and incorporate e-consultation as part of a manageable clinical workflow.

## Introduction

### Background

The growth and ageing of the global population combined with increased expectations place enormous pressures on primary health care. Greater use of technology is seen as a partial solution to the complex challenges of delivering health care to an increasing and ageing population with more chronic disease. This is reflected in health policy in the United Kingdom, the United States, and elsewhere [1]. Technology-supported consultations provide more flexible, though different, style of the clinician-patient relationship. However, adoption has been a challenge [2], and there is limited evidence of benefit [3,4].

The United Kingdom has taken a strong interest in using technology to deliver care [5], mainly driven by the increased cost of emergency administrations. Between 2012 and 2013, there were 5.3 million emergency admissions to UK hospitals, at a cost of approximately £12.5 billion representing a 47% increase over the previous 15 years [6]. These increases have led to growing interest as to whether remote care reduces what is considered unnecessary doctor’s appointments or avoidable hospital admissions. However, to be commissioned and mainstreamed into everyday practice, an innovation must show that it can provide significant system-level advantages effectively providing *more for less*. For example, one of the worlds’ largest remote care trials, a whole system demonstrator project saw improvement in patients’ quality of life [7-9]. Telemedicine has also shown benefits in terms of health outcomes, hospital admission, and in terms of cost-effectiveness [10-12].

In this study, we focus on electronic consultations (e-consultations) situated within primary care. Remote care comes in many forms, including telephone, video, text messaging, email consultations, Web-based portals for prescription orders, appointment booking, and patient access to online health records, or any combinations of all these [13], recognizing that research in this area is heterogeneous [14]. We have excluded telemedicine and telemonitoring and generally specialist-based care that focus on the long-term management of chronic conditions.

E-consultations are feasible, and reliable, and convenient [15], although in common with other digital innovation challenging to implement [16]. Despite the growing use of computerized medical records [17], it has been challenging to incorporate e-consultations into clinical workflow [18,19]. To date, trials show little or no significant difference between usual care and intervention groups in terms of clinical outcomes [20].

### Objectives

The aim of this review was to assess the evidence of delivering e-consultations using secure email, messaging or video links in primary care. The objectives were as follows: (1) understand how e-consultations affect patients’ access to services, their frequency of use and satisfaction, and any impact on health outcomes; (2) investigate professional and workforce issues, including potential changes in workload or flow (actual and perceived) and barriers to use; and (3) identify possible organizational or technology barriers and solutions to implementation.

## Methods

### Design

**Figure 1:**
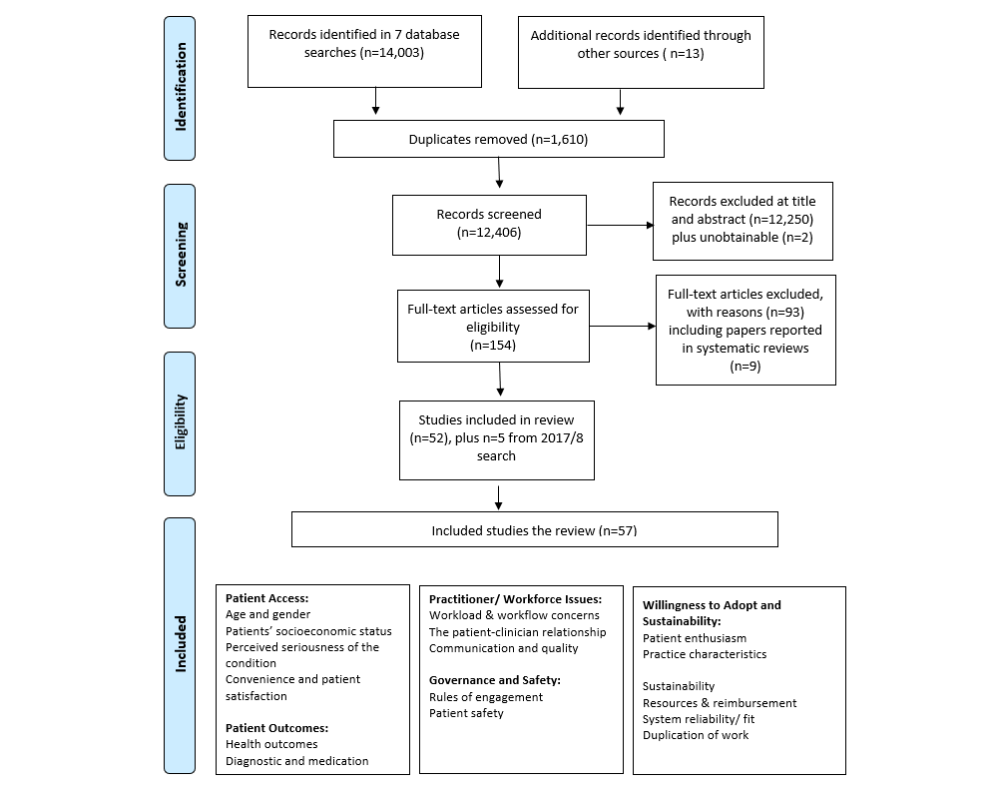
Preferred Reporting Items for Systematic Reviews and Meta-Analyses.

This systematic review follows Preferred Reporting Items for Systematic Reviews and Meta-Analyses [21] guidelines (Figure 1). The study aims were structured using the population, intervention, comparator, and outcome format [22]. The study *population* was defined as users or nonusers of e-consultation services, including both patients and carers and clinicians as well as support staff in primary care. The *intervention* related to synchronous or asynchronous e-consultation service used in primary care. Any *comparison* was used, including usual care. Several *outcomes* were identified including the following:

1. Patient(s): changes to service use including access to services (by specific patient groups, disorder or attributes of the user, frequency of attendance, and satisfaction), and impact on health outcomes.
2. Professional or workforce: workload and barrier to e-consultation implementation, impact on professional identity, consultation or revisit rates, and finally (if the information is available) quality and safety (ie, complaint numbers).

The protocol was registered on PROSPERO, the international database of systematic reviews, registration number CRD42015019152.

### Information Sources and Searches

Advanced searches were performed across a range of bibliographic databases, including, the Cochrane Library, general medical databases (MEDLINE, EMBASE, CINAHL, via EBSCO platform), PsycINFO, EconLit, and Web of Science. A search was performed in the database OpenGrey for unpublished material.

Search strings were developed according to the index terms (Medical Subject Headings [MeSH] for MEDLINE) of each database together with keywords within the title or abstract using Boolean searches (AND, OR) with truncation and wildcard functions used (Multimedia Appendix 1).

This is an emergent and developing area, so recently published research was of key interest. We searched the literature from January 1, 1999, to March 1, 2017. No limits were placed on the evidence type (type of document, ie, systematic review), country of origin, or language of literature. Search results were exported into EndNote (v7.2.1). The search yielded 14,016 references, of which 1610 were duplicates and 12,406 were screened.

### Setting and Participants

The systematic review focused on primary care and ambulatory care settings. Our principal participants in this study were patients and their family, caregivers (users and nonusers of e-consultations), and health care professionals (clinicians, allied health professionals, practice support staff, and managers). The technology is also relevant and was included in this review, focusing on current implementation, design, and the Information and Technology infrastructure underpinning e-consultations.

### Eligibility Criteria

Search results were checked against the predefined inclusion and exclusion criterion (see Multimedia Appendix 2 for excluded studies). The inclusion criteria were based on the following: (1) a range of health care conditions, including any long-term chronic conditions managed in primary care (diabetes and hypertension) or routine conditions (skin conditions and sleep issues); (2) any asynchronous and synchronous use of emails and visual or video technologies (eg, Skype) used by both patients, carers and health care professionals in the e-consultations; and (3) no limitations were placed on the type of study (randomized controlled trial [RCT], qualitative, quantitative, and economic impact); however, study protocols were excluded as they do not contain original outcome data or review evidence.

Exclusions were studies focusing on telephone use alone (without the use of email, video or messaging) and any experimental studies which fail to provide specific outcomes measures or reported quality measures for service evaluation purposes only (eg, National Health Service Information Centre Quality and Outcomes Framework summary data). Finally, studies were excluded if they reported the use of medical records, email or telephone to recruit participants to research projects. This review only includes studies that performed e-consultations with primary care staff, with services performed in other settings (the community, secondary, or tertiary care) being excluded. Other studies were excluded if they focused on health promotion or education tools, which was not the primary focus of this review. Specifically, we were interested in e-consultations impact on access and health outcomes related to an illness event, rather than on long-term preventative strategies. Budgetary constraints excluded the authors from including studies that needed to be translated. Finally, to avoid possible bias and overreporting, studies were excluded if their results were already reported on in included review article [23]. All included studies were required to involve the patient in the e-consultation with their primary care provider. As such, provider-to-provider interactions were excluded from this review.

### Data Selection

Evidence was sourced and retrieved by members of the research team (FM and YL). Results from searches were stored electronically. An initial screening of titles and abstracts was independently conducted by 2 team members (YL and FM). Inclusion queries were resolved through discussion at team meetings. Inclusion decisions were recorded using EndNote (v7.2.1). Further exclusions occurred once full texts were retrieved and when papers failed to meet the inclusion criteria or were a poor fit.

### Data Extraction

Independent duel data extraction was undertaken by 2 researchers using a predesigned data extraction form (DEF) reflecting the core objectives of the study, including aims and objectives, study design, setting, type of e-consultation, outcome measures, comparator groups, and key findings. Data extracted also focused on a range of clinical outcomes (such as hemoglobin HbA_1c_ and blood pressure), behavioral outcomes (patient-clinician interaction, perceptions, acceptance, and system use), and organizational issues (such as functionality, usability, cost, and workflow). The DEF aimed to assist the authors to consistently retrieve the core contents of each study and aid in the organization of material before analysis.

### Data Analysis and Quality Assessment

The analysis was executed in several stages. The first stage was the identification of the themes arising from the literature. The themes were developed over a series of meetings when the researchers clustered the results into higher order categories that seem to have coherence when summarized together. The aim of the clustering was to devolve a large and varied number of results into a smaller number of more easily understood, salient issues. The analysis was supported using a 3-stage thematic analysis process previously used [24,25] and guided by the Mayring framework [26]. The second stage included the assessment of evidence quality. Finally, themes were grouped against each of the research objectives to build up a comprehensive overview of the evidence. The analysis was undertaken by FM and JH with periodic input from the wider team.

### Critical Appraisal

Studies based on qualitative, quantitative, and mixed methods designs were subject to critical appraisal, using the Mixed Methods Appraisal Tool (MMAT 2011 version) [27,28]. The MMAT tool uses criteria scored from 0% to 25%, with the overall score being 100. The interrater reliability of the MMAT was 0.94 [27]. No quality threshold was imposed, but caution was used to not overemphasize the contribution of evidence which had a low score (50% and less; n=7 papers, 25%). In reporting findings, greater emphasis has been placed on the literature with a higher MMAT score (>50% and above; n=41). For this work to be transparent, we have reported the MMAT score table (see Multimedia Appendix 3).

## Results

### Study Characteristics

A total of 57 studies were included in the review (n=57), including evidence from a range of countries, the United States (n=30) and the United Kingdom (n=13), with the remaining from Australia (n=3), Sweden (n=3), Finland (n=3), Canada (n=3), Denmark (n=1), and Italy (n=1), enabling greater ability for the findings to be generalizable (See Multimedia Appendix 4).

A variety of study designs were used, although the majority employed quantitative methods including descriptive designs such as surveys, and analysis of service frequency data (n=22) [29-50], quasi-experimental, cohort, or cross-sectional designs (n=10) [51-60], or RCTs (n=2) [61,62]. There was also a range of qualitative study designs using case studies, interviews, and focus groups (n=13) [63-75]. Only 6 studies had a mixed method design [76-81]. A total of 4 review findings were included [20,82-84].

A total of 5 overarching themes were identified across the literature: patient access, patient outcomes, workforce issues, governance and safety, and factors that impact on willingness to adopt and sustainability.

### Patient Access

#### Age and Gender

The sociodemographics of patients using e-consultations was mixed. Users of e-consultations [29,38,81,82] and secure messaging [40,55] were primarily women [29,38,40,41,43,55,81,82] who used these services during working hours [29], presumably because of issues of convenience [41] in terms of organizing care or treatment for dependents (young children or older relatives) [30]. However, the evidence is far from conclusive, as 1 study found no statistical difference between genders [58], and another study found that more men (59/87) than women used the service (28/87) [54]. The mean age of e-consultation users also varies. Some studies report prevalent users as being younger (45.9 vs 50.3 years, *P*<.01) [58], some as being 31-49 years (63/87, 77%) [54,82], middle-aged (50-65 years) [55], or over 60 years of age [43].

A study comparing patient characteristics receiving face-to-face or e-consultation in primary care (sinusitis and urinary tract infection [UTI]) found older people (≥65 years) to be less likely to use e-consultations (sinusitis, 28/475, 5.8%; UTI, 9/99, 9%, *P*<.001) [38]. In a similar study, age (over >65) was also associated with being less likely to use secure messaging (odds ratio [OR] 0.65, 95% CI 0.59-0.71) [55]. Early evaluation of e-consultations in one clinic suggested older patients found the concept of e-consultations confusing [81]. In contrast, a systematic review in 2014 suggests concerns about older patients being confused by them may be unjustified, and benefit could be gained if offered the right support [82].

#### Patients’ Socioeconomic Status

Direct measures of socioeconomic status or failure to have health insurance, which we took as an indirect measure of socioeconomic status, were associated with limited affordability and access to emerging technologies [71]. Socioeconomically disadvantaged patients or those with poorer self-reported health were less likely to express an interest in communicating about their care using email or the internet [35]. In addition, patients who used email to communicate with their clinician were significantly associated with a higher annual family income (*P*=.007; >US $70,000) [34,43]. This group was reported to communicate with their clinician twice as much as those on lower incomes (<US $10,000-29,999) [34]. Moreover, a study investigating the characteristics of e-consultation patients found a high number of employed patients (for conditions such as sinusitis, 355/475, 74.7%; or UTI, 59/99, 60%; *P*<.001), suggesting out-of-office access is important for those in work [38].

In contrast, 1 study suggests the lack of medical insurance increased the odds of using 2-way visual and audible contact with health providers (OR 0.83, 95% CI 0.72-0.97) [41]. The cost of e-consultations for patients (email via a portal) varies between US $35 [29] and US $39 [39]. Earlier work found there may be a cost threshold, with 60.1% (149/248) of patients willing to pay up to US $10 or more per year. Only 31.0% (77/248) of patients were willing to pay up more—up to US $50 or more per year for secure email contact [31]. Willingness to pay did not differ by age (*P*=.06) [31].

#### Perceived Seriousness of the Condition, Convenience, and Patient Satisfaction

Patients reported using e-consultations when they did not perceive that a face-to-face consultation as warranted, even if conditions were chronic and long term such as diabetes and hypertension [29,57,79], or in cases where symptoms were routine or nonurgent, such as skin conditions, low-level pain, sleep issues, hemorrhoids, coughs, or sinusitis [29,48,79,81,83]. Unlike other studies, email contents analysis in 1 study suggests emails are useful when patients want to request information (symptom updates) or simple provider action (referrals, medications, treatments, or test result information) [63]. This suggests e-consultation [67,83] and online primary care visits [29] offer a convenient means through which to manage low-risk, nonurgent health concerns.

Differences also emerged when using technology to receive test results. Although many patients were willing to use email to obtain test results for cholesterol (1045/1229, 85.02%), less were willing to use this mode of contact for more serious conditions such as receiving a brain computed tomography scan test result (725/1229, 58.99%) [34]. Perceived seriousness also impacted on the mode of communication, with patients reporting favorable attitudes toward email but not text message or a Web page for the delivery of blood test results [44].

Convenience was the primary reported reason for choosing an e-consultation by patients across multiple studies [35,38,41,45,48,67,79,83]. Patient satisfaction [32,51,59,66,70] with immediate care received was increased [81] in the short term at 6 months [52]. Studies exploring the possible long-term impact of e-consultations over face-to-face encounters reported similar findings [40,52]. One study found no significant difference in the 30-day adjusted visit frequency at follow-up (2.35 visits per year before and 2.35 after portal messaging, *P*=.93) [40]. The subgroup analysis at 1 year of follow-up found an adjusted nonsignificant decrease of 0.1 visits per year (2.44 visits per year before the first message) and 2.34 after (*P*=.14) [40].

Timeliness of responses was important to patients using email [33,74,81] and was associated with satisfaction [84]. Patients had high expectations regarding the timeliness of responses for various Web-based services. Almost all patients in 1 study (2011/2260, 88.98%) expected a reply from email messages from clinicians within 24 hours, and 67.96% (1536/2260) expected responses or access to laboratory results within a 24-hour period [34]. More than 50% of patients expected a reply within 8 hours [34] and preferably the same day [74].

A range of studies found specific advantages to using e-consultations including improved access to care [66,70,83], both in the delivery of care outside of standard working hours [73] and care delivery to remote areas, time saved [32,36,45,73], and cost-saving including lost wages [73]. One evaluation study, of joint teleconsultations among general practitioners (GPs), specialists, and patients, found cost-saving for patients between €1,000.06 and €2700.50 by patients avoiding travel to emergency departments and for in-clinic visits or diagnostic examinations [50]. Finally, video and email consultations provide both patients and clinicians with opportunities to learn about health conditions and their management, through information and image sharing [65,74], offering the potential for more active patient engagement in the care process [52,63,82].

Joint e-consultations among GPs, specialists, and patients resulted in significantly higher levels of patient satisfaction (mean difference 0.33 scale points, 95% CI 0.23-0.43, *P*<.001) [62]. Satisfaction was also associated with a reduction of distance travelled [38] (average decrease of 170 kms) [32] or 1-way distance saved per patient (average 65 miles) [36]. Not surprisingly, greater e-consultation use was associated with the winter months [38], especially for patients (and families) using video consultations in rural and remote communities [73].

### Patient Outcomes

There is a lack of good quality evidence demonstrating positive patient outcomes from e-consultations because of the heterogeneity of existing evidence making an accurate assessment of benefits difficult [20]. In addition, there are limitations as to the longevity of follow-up data in trial material, again limiting the generalizability of any findings [20]. There were, however, several areas of potential benefit highlighted. Survey evidence suggests how telemedicine was as good as or even better than face-to-face consultation concerning the explanation of care to patients [32]. Email consultations were also shown to be clinically feasible in terms of diagnostic accuracy [84].

E-consultations may also play a role in the management of symptoms [51,57]. A study focusing on the management of hypertension in rural areas, using videoconferencing, found that the intervention group had a higher proportion of patients with blood pressure within treatment goals (systolic blood pressure, 140 mmHg; diastolic blood pressure, 90 mmHg), both at baseline and at follow-up, compared with a comparison group [57]. The intervention group was shown to have a higher probability of meeting their target blood pressure goal (OR 2.7, 95% CI 1.4-5.2) over the comparison group [57]. The quality of physical examinations in e-consultations was significantly worse regarding effectiveness (2.3 vs 4.9 for the face-to-face visit, *P*<.001), but history taking and therapeutic effectiveness were not significantly different [59].

### Workforce

Several studies report clinicians’ reluctance to use email with their patients because of increased workload concerns [37,40,46,84]. Clinicians reported improved efficiencies as email or secure messaging was described as taking little additional time [70] and encouraged care access [79]. However, as time is cumulative, even small additions, for example, between 2 and 6 min per email consultation [84], may lengthen the working day [70,76]. A quasi-experimental study reported how offering access to visit notes or email contact to patients was actually easier than expected and resulted in no change in the volume of messaging from patients [51]. Indeed, few clinicians reported longer visits (0%-5%) or more time answering patients’ questions outside of face-to-face visits (0%-8%) [51]. Practice size has little effect on the overall workload [51]. Similarly, an evaluation of an email service found email services did not have any adverse time implications [66]. As such, practice partners were satisfied that the service worked effectively and did not negatively impact their day-to-day workload [66].

A retrospective cohort study of patients (n=2357) using electronic messaging (both secure messages and e-consultations) via a portal found, after the first message surge, no significant visit frequency differences (mean 2.35 annual visits per patient both before and after the first message, *P*=.93) [40]. Subgroup analysis indicated no significant change in the frequency of visits between high messaging users, or for those who had used messaging for longer. In other studies, e-consultations were found not to reduce telephone consultations [79] or number of office visits [70]. Evidence focusing on return visits to primary care found no significant differences in rates of early return visits for the same reason (e-consultations 20.2%, 46/228; face-to-face 19.6%, 98/500; *P*=.86) [58]. Similarly, a pilot study found less than <10% of patients who had an e-consultation (*similar to email*) required a follow-up face-to-face appointment [78]. Only the presence of moderate or more comorbidities was a significant predictor (OR 1.95, 95% CI 1.20-3.17; *P*<.01) relating to return visits for the same reason [58]. A small questionnaire to determine the feasibility of conducting follow-up visits using videoconferencing compared with face-to-face visits reported no significant difference in either group at 6 months [52]. Overall, findings from multiple studies suggest the use of e-consultations may complement in-person delivery (or could be a useful adjunct) to routine care [68,79,84], but this is reliant on the seriousness or risks associated with specific health conditions [58,68,79].

#### The Patient-Clinician Relationship

E-consultation was reported to impact on the patient-clinician relationship. The quality and safety of communication between groups may be affected as well as the interpersonal relationship (both positively and negatively). Access to physician notes and electronic messaging impacted on who initiated the direction of contact [70] and quality of the clinician and patient communication (content and tone) [51,63,73,79,83,84]. The ability to immediately exchange information (in a timely manner either asynchronous or synchronously) was reported to potentially improve the therapeutic relationship [84]. Clinicians felt patients’ access to visit notes and electronic messaging strengthened their relationship with some patients because of a sense of enhanced trust, transparency, communication, and shared decision making [51,79]. Email exchange was also viewed as a useful tool to enable patients to express individual concerns and building a partnership, which was supportive and patient centered [63,83]. Video consultations in remote areas were also seen as an effective way to maximize home support, bring comfort to users in their own homes, and bring providers and families together from various regions [73].

In contrast, there were concerns about how e-consultations might negatively impact on the clinician-patient relationship [68]. These concerns include the need for professionals to communicate using nontechnical language [69] and their need to manage multiple tasks simultaneously (such as recording information), which might impact on the perceived engagement and attentiveness of the clinician in the Web-based interaction [75]. Indeed, in circumstances where nurses were present with clinicians in the e-consultation, clinicians themselves sometimes felt like outsiders, as the nurse and patient were better able to form a mutual bond via nonverbal communication and empathetic skills (such as maintaining eye contact) [75].

### Governance and Safety

Within this review, governance, quality, and safety issues emerged in various forms, but not widely researched [39]. Only 1 study, a retrospective analysis of secure messaging and e-consultations was undertaken to assess the potential risk of time-sensitive symptoms, such as chest pain or dyspnea [39]. Only 6 hospitalizations were related to a previous secure message (0.09% of secure messages), and 2 hospitalizations were related to previous e-consultations (0.2% of e-consultations, 2/892) [39]. Quality emerged in terms of the mode of care delivery either in terms of offering patients’ information which impacts on their future service use, such as offering information which decreases the need for face-to-face encounters [60], enabling further opportunities to identify new problems during e-consultations [36] or raising perceptions of medicolegal liability [79].

Clinicians also raised concerns related to the lack of guidance about the *rules of engagement* [67], such as if an email is left answered [79] or level of confidence about taking medical history via e-consultations rather than face-to-face [52]. In response to the lack of guidance, GPs and patients have introduced their own rules of contact. These rules were not comprehensive and did not cover all eventualities [67]. Lack of formal practices and guidance was a recurring issue across the evidence [74,76,83]. A final concern is whether instructions through email can be adequately understood and correctly acted upon as intended by the sender [20,79] and whether some questions were appropriate for discussion via email [74].

### Factors That Impact on Willingness to Adopt and Sustainability

Willingness to use technologies can be broadly divided into 2 related themes: the patient perspective and professional or organizational perspective. Low response rates among users were prevalent across studies [37,56,76], indicating differences in use depending on the level of experience between first users and those who are more experienced [36,46,76,81].

Patient enthusiasm was often dependent on their previous experience of using technology to manage their health [56]. In a longitudinal study comparing pre and post attitudinal changes to e-consultation found that first-time users were more likely to have a positive view, whereas experienced users were more negative (*P*=.025), suggesting patient use may tail off over time [54]. Other factors impact on patients’ willingness to try e-consultations, including perceived severity of the condition (minor complaints) [79] and the actual mode of communication (secure email, direct access to records or laboratory results) [44].

General practices’ willingness to adopt may also manifest in terms of the actual characteristics of the general practice (size and location) [71], with smaller practices in more deprived areas being less likely to use email [77]. Clinicians working in group practices were reported to be more in favor of using video technology for consultations [49].

In terms of sustainability, e-consultation may have repercussions in respect of further work across settings. A pilot mixed methods study found that specialist consultation requests made into primary care clinicians [78] resulted in GPs being asked to offer more patient advice, order diagnostic tests, or commence a new course of treatment [78]. Other work has echoed this potential service *push* to other health care providers with teleconsultations, resulting in a small number of additional diagnostic examinations (n=8) and hospitalizations (n=6) [50]. Similarly, an RCT examining whether e-consultations (called virtual outreach in the study) among GPs, specialists, and patients would reduce follow-up appointments found more e-consultation patients than the standard group being offered a follow-up appointment (502/971, 51.6%, vs 400/971, 41.1%; OR 1.52, 95% CI 1.27-1.82; *P*<.001) [62]. There was, however, variability associated with rates of follow-up according to specialty and site [62].

With regard to implementation and sustainability, there is limited evidence available about the cost-effectiveness of e-consultations, but the high cost of buying telemedicine equipment [46] and expense of implementing this technology is a concern for health care professionals [61].

Costs of clinicians’ time to support joint consultations were unlikely to be offset against subsequent savings to health care services in the short term [61]. The total use of UK health care (NHS) resources over 6 months suggests that the overall mean cost per patient is significantly higher in the joint consultation group than the standard outpatient group by approximately £100 [61]. The significant reduction in tests and investigations in the joint consultation group resulted only in small cost reduction *downstream* [61]. Similarly, other studies recommend future long-term follow-up (over 6 months) to determine downstream outcomes and full evaluation of cost-effectiveness [62].

Delays in service delivery was also an additional concern with the provision of out-of-hours services. A small study assessing delayed response to patients’ secure email messages (messages not opened after 12 hours or nonresponse after 36 hours) found both kinds of delays were higher on weekends (*P*<.001) (Friday-Sunday) [40]. Delay was more likely to be experienced by patients aged over 50 years (605/2357, 25.66% delayed; *P*=.013) [40]. The study suggests that these delays could be addressed by automatically rerouting messages to a 24-hour staffed support service or another mechanism to manage this after-hour workflow [40]. Provision of logistical support for a range of e-consultation methods may, therefore, be significant to enable long-term and efficient implementation of systems in primary care [62]. In addition, in 1 study, facilities which offered user support for those wanting secure messaging were found to have higher rates of adoption (2.13%) over other providers (1.52%; *P*=.006) [56].

Other notable barriers to implementation include commissioners’ incentives (or direction of cost) for the introduction of remote services [65], the impact of size and location of practices [71], and organizational resistance [59,77]. From the provider’s perspective, a mixed method study suggests email communication could be embedded into everyday practice and be remunerated similarly to usual clinic time, thereby potentially offering a new structure of care [79]. The direction of cost is illustrated in 1 study exploring the experience of Greek health care providers and their patients with the introduction of an e-consultation service [65]. The study found that there was no incentive for the health care system to introduce e-consultations as often patients incurred the cost of their own travel to the mainland for health care [65]. Implementation may also be influenced by whether e-consultations in practice were resource- or reimbursement**-**driven [37,71,81].

The final sustainability consideration is system-level fit, the extent that e-consultations can integrate into existing services and the scalability of implementing this technology.

Scottish research on the uptake of an electronic clinical communication system reported that although the current system was beneficial, issues around system reliability, incompatibility of systems, and duplication of data hindered widespread uptake [45]. The main perceived barrier to adoption were views about the instability of computer networks across the region [45]. Technology design was also seen as critical in relation to ease of use and functionality for both patients and health care professionals [36,46,76,81] and can be directly linked to uptake or adoption [76]. Functionality is also important to clinicians [46,81]. This emerged in reference to possible technical failure, level of previous and current training needs, experiences of technology use (both positive and negative), and the condition, state and age of the available technology [61].

#### Mixed Methods Appraisal Tool Results

The overall MMAT study quality was moderate, with only 11 studies identified as excellent (100%). However, use of the MMAT, aided both description and appraisal of studies, helping to highlight the need for robust and larger trials as well as to fully explore the level of risk, both real and perceived [58,79].

As previously mentioned, generalizability of some studies was limited [46,55,78] in many cases by low participant numbers [37,52,68] or single or low number of study sites [34,35,37,40,43,51,66]. Owing to the heterogeneity of (OR and hazards ratio) measured outcomes across studies, the study team decided not to conduct a meta-analysis, as this may have resulted in a misrepresentation of the data.

## Discussion

A total of 5 themes emerged which addressed our review objectives. These themes were patient access, patient health outcomes, workforce issues, governance and safety, and finally willingness to adopt and sustainability of e-consultations.

### Patient Access

In understanding how e-consultations affect patients’ access to services, there is evidence to suggest that e-consultations work well for some patient groups but not for others impacting on access, with the elderly and the poor less likely to use these services [36,39,56,72]. As such, there was a disparity between different users and under what circumstances patients are more willing to use e-consultations systems and why.

### Patient Health Outcomes

There was also a lack of evidence of whether patient health outcomes improve with e-consultations [20]. Indeed, a potential limitation to this study is the dearth of studies reporting health outcomes from e-consultations. As such, there is a need for further high-quality studies to fully evaluate the usefulness of e-consultations in primary care, especially on how patient outcomes are affected and the long-term impact of e-consultations on the patient-clinician interactions.

### Workforce Issues

In investigating professional and workforce issues, evidence suggests that e-consultations may increase patient expectations of care delivery [34] and complement existing in-person care [68,79,84]. There were, however, differences in the perceived rise of work demand for clinicians and the actual manifestation of raised workloads reported in studies, with clinicians reporting little additional time [70] or volume of messaging from patients [51].

E-consultations may also impact on the patient-clinician relationship in terms of changing the quality of the communication [51,63,73,79,83,84], either by fostering an enhanced sense of trust or transparency in communication [51,79] or highlighting communication deficiencies regarding the interpersonal skills needed to manage Web-based interactions [69,75].

### Governance and Safety

The review highlights the lack of evidence or guidance about any rules of engagement for technology consultations and the challenges this presents to patient safety [66,74,76-78,83,85]. An appropriate consultative discussion to clarify *terms and conditions* and guidance may enhance professionals’ confidence in using these systems and positively impact on implementation and sustainability of e-consultation.

Further research is also needed to explore the value and perceived benefit of care provision beyond core working hours (8 am to 6.30 pm, Monday to Friday). Expectations of timeliness arising from this review may lead to pressures in other areas of the health care system, such as secondary care services (accident and emergency providers). Despite the challenges of providing comprehensive care coverage to meet changing demographics and health care demands, early research does suggest the need to manage and deliver care outside of traditional infrastructures [86].

Consideration also needs to be given to quality and safety concerns, especially in relation to the accuracy of e-consultations diagnoses, or whether differences emerge in the quality and safety of prescribing (face-to-face vs e-consultation), including by whom—physician or advanced practitioner [87,88].

### Willingness to Adopt and Sustainability

Finally, identifying possible organizational or technological issues related to the implementation of e-consultations found little evidence of studies being sustainable in the longer term (up to 1 year) [40,52]. Therefore, consideration needs to be given to whether these systems are only useful at specific time points in the patient journey, for example, newly diagnosed patients with specific conditions, or whether e-consultations could be more broadly applied across conditions. Indeed, studies into a willingness to pay were also underrepresented [61], and caution is reported in other studies suggesting the need to adequately fund organizations before establishing video consultation as routine in general practice [49]. This perhaps suggests a need for further research, to capture longer term economic data related to e-consultation, an important consideration for any provider considering implementation [40,48,89]. Adopting e-consultations may also enable greater communication between clinicians [71], across specialist and primary care [73,78], and a broader range of geographical urban and rural areas [33,71,82].

### Strengths and Limitations

In a fast-moving field, it is impossible for reviews to always include the latest developments, and some of these may be commercialized without publication. In addition, we faced the challenge of appraising if recent studies carried out in outpatient clinics are relevant to primary care [90-94]. Finally, in conducting this review, we also appreciate there are some technology and infrastructure differences between the countries, including limitations in using emails to communicate with patients. This may also have limited the reporting of results, especially if some studies were not translatable into English.

### Conclusions

E-consultations are intended to address the growing demand for care from general practice. Policies and new funding opportunities that support innovative ways of care delivery may encourage a cultural shift in how patients interact with professionals and manage their own care, while also shaping the way primary care professionals use and manage technology in their practice to provide safe and efficient care.

There are 3 key messages identified from this review which may be considered important in the future developments of e-consultations. First, the review provides some insight into who, why, and when specific patient groups may be disproportionally disadvantaged or advantaged by using Web-based systems. Second, consideration needs to be given to providing a better understanding of patients’ views about privacy and security of their data, so patient privacy and confidentiality are ensured. This may include exploring patients’ views across different health conditions or time points, as perceived seriousness of their conditions is one key factor influencing willingness to consult electronically. Finally, issues impacting on professional’s use of and perceptions of e-consultation may also be a limiting factor in terms of adoption. Fears of extra workload, expectations of quick response time, insufficient guidelines or training about the *rules of online engagement,* and effective communication strategies were all factors impacting on use.

Our review suggests that e-consultations may improve aspects of care delivery, but there remains uncertainty about which potential users to target. Improved e-implementation is a high priority, as well as the further work needed to develop innovations which support equitable primary care access and delivery.

## Appendix

Multimedia Appendix 1 Resubmission - Example Search String.

Multimedia Appendix 2 Resubmission - Inclusion and Exclusions.

Multimedia Appendix 3 Resubmission - MMAT Quality Appraisal.

Multimedia Appendix 4 Resubmission - Evidence Tables.

## Abbreviations

DEF: data extraction form
e-consultation: electronic consultation
GPs: general practitioners
MMAT: Mixed Methods Appraisal Tool
OR: odds ratio
RCT: randomized controlled trial
UTI: urinary tract infection
