# Supplementary material for: Electronic Consultation in Primary Care Between Providers and Patients: Systematic Review
Source: JMIR Med Inform. 2019 Dec 3;7(4):e13042. doi: 10.2196/13042 (PMC6918214; doi:10.2196/13042)
Supplement: Multimedia Appendix 1 [file medinform_v7i4e13042_app1.docx]

**Multimedia Appendix 1 – Example Search String**

**Interface – EBSCOhost Research Database**

**Search Screen – Advanced Search**

**Database – Medline**

| **Web** | |
| --- | --- |
| **1** | (online OR web* OR internet) N4 (consult* OR service* OR intervention* OR therap* OR treatment* OR counsel*) ab.ti |
| **2** | (web* OR internet OR www OR electronic* OR online) N5 (messag* OR communicat* OR transmit* OR transfer* OR send* OR deliver* OR feedback OR letter* OR interactive* OR input* OR forum OR appointment* OR booking* OR referral* OR consult* OR prescri*) ab.ti |
| **3.** | OR/1-2 |
| **4.** | NOT telephone |
| **Email** | |
| **5.** | ((MH "Caregivers") OR (patient* OR carer* OR consumer*)) N5 ((MH "Computer Communication Networks") |
| **6.** | (MH "Electronic Mail") OR (electronic mail* OR email* OR e-mail* OR web mail* OR webmail* OR internet mail* OR messag*)) |
| **7.** | (MH "Mobile Applications") OR “mobile app*”.ti.ab |
| **8.** | OR/5-7 |
| **9.** | NOT telephone |
| **e-consultation** | |
| **10.** | (e-communication* OR e-consult* OR e-visit* OR e-referral* OR e-booking*) OR (ecommunication* OR econsult* OR evisit* OR ereferral* OR ebooking*)ab.ti |
| **11.** | (MH "Remote Consultation") OR “e*consultation” ab.ti |
| **12.** | AB "remote consultation" OR TI "remote consultation" ab.ti |
| **13.** | AB “remote communicat*” OR TI “remote communicat*” ab.ti |
| **14.** | “remote access*”ti.ab |
| **15.** | (MH referral and consultation) |
| **16.** | OR 11-15 |
| **17.** | NOT telephone |
| **Skype/ Videoconferencing** | |
| **18.** | (MH "Telemedicine/EC/MT/OG/TD/UT") (Economics, methods, organisation, trends and utilisation) |
| **19.** | "teleconsultation" OR "teleconsultation" OR TI "teleconsultation" ab.ti |
| **20.** | (MH "Videoconferencing") OR |
| **21.** | (MH "Computer Communication Networks") |
| **22.** | "teleconsult*".ti.ab |
| **23.** | (MH telemetry) |
| **24.** | OR/18-24 |
| **25.** | NOT “telephone” |
| **Interface** | |
| **26.** | (MH "Attitude to Computers") |
| **27.** | (MH "User-Computer Interface") |
| **28.** | OR/26-27 |
| **Primary Care** | |
| **29.** | (MH "General Practice") OR (MH "General Practitioners") OR (MH "Family Practice") OR (MH "Primary Health Care") OR (MH “ambulatory care”) OR (MH “community health services”) |
| **30.** | “primary care” OR “community-based provider*” ab.ti |
| **31.** | OR/ 29-30 |
| **32.** | OR/ 4-9 (Web & Email) |
| **33.** | OR/17-25 (e-consultation & Skype/ video) |
| **34.** | OR 32-33 AND 31 (primary care) (Results 1) |
| **35.** | 34 AND 28 (results 1 & interface)(Results 2) |
