# Supplementary material for: Electronic Consultation in Primary Care Between Providers and Patients: Systematic Review
Source: JMIR Med Inform. 2019 Dec 3;7(4):e13042. doi: 10.2196/13042 (PMC6918214; doi:10.2196/13042)
Supplement: Multimedia Appendix 2 [file medinform_v7i4e13042_app2.docx]

**Multimedia Appendix 2 - Exclusion Studies**

| No. | Author/Year | Title | Reason Code |
| --- | --- | --- | --- |
| 1 | Abanes J & Adams S (2014) | Using a web-based patient-provider messaging system to enhance patient satisfaction among active duty sailors and Marines seen in the psychiatric outpatient clinic: a pilot study | 2 |
| 2 | Agha Z et al. (2002) | Cost effectiveness of telemedicine for the delivery of outpatient pulmonary care to a rural population | 2 |
| 3 | Also O et al. (2012) | Mobile health IT: the effect of user interface and form factor on doctor-patient communication | 2,4 |
| 4 | Armstrong, AW (2010) | Evaluation and comparison of store-and-forward teledermatology applications | 2 |
| 5 | Atherton H et al. (2012) | Email for the coordination of healthcare appointments and attendance reminders | 2 |
| 6 | Bangs I et al. (2002) | An integrated nursing and telemedicine approach to vascular care | 7 |
| 7 | Bardach N et al. (2009) | Evolving health information technology and the timely availability of visit diagnoses from ambulatory visits: a natural experiment in an integrated delivery system | 2 |
| 8 | Barry N et al. (2003) | Implementation of videoconferencing to support a managed clinical network in Scotland: lessons learned during the first 18 months | 2 |
| 9 | Barton P L et al. (2007) | Specialist physicians' knowledge and beliefs about telemedicine: a comparison of users and nonusers of the technology | 2 |
| 10 | Bergmo, T. S. and S. C. Wangberg (2007). | Patients' willingness to pay for electronic communication with their general practitioner. | 3 |
| 11 | Bergus G et al. | Email teleconsultations: well formulated clinical referrals reduce the need for clinic consultation | 2 |
| 12 | Brooks, R. G. and N. Menachemi (2006) | Physicians' use of email with patients: factors influencing electronic communication and adherence to best practices. | 3 |
| 13 | Bowater M (2001) | The experience of a rural general practitioner using videoconferencing for telemedicine | 2 |
| 14 | Bujnowska-Fedak M M et al. (2000) | System of telemedicine services designed for family doctors' practices | 7 |
| 15 | Campbell J D et al. (2001) | Introducing telemedicine technology to rural physicians and settings | 2 |
| 16 | Casey, M. et al. (2013) | Implementing transnational telemedicine solutions: A connected health project in rural and remote areas of six Northern Periphery countries | 2 |
| 17 | Christensen, T & Grimsmo, A (2005) | Development of functional requirements for electronic health communication: preliminary results from the ELIN project | 3 |
| 18 | Cicolini, G. et al. (2014) | Efficacy of a nurse-led email reminder program for cardiovascular prevention risk reduction in hypertensive patients: A randomized controlled trial | 2 |
| 19 | Coelho, K. R. (2011) | Identifying telemedicine services to improve access to specialty care for the underserved in the San Francisco safety net | 2 |
| 20 | Couchman, G. R. Forjuoh, S. N. Rascoe, T. G. (2001) | E-mail communications in family practice: what do patients expect? | 3 |
| 21 | Cole-Lewis H, Kershaw T (2010) | Text messaging as a tool for behavior change in disease prevention and management | 2 |
| 22 | Dambha, H (2013) | Use of email for consulting with patients in general practice | 3 |
| 23 | De Las Cuevas, C. et al. (2006) | Randomized clinical trial of telepsychiatry through videoconference versus face-to-face conventional psychiatric treatment | 2 |
| 24 | DeJong, C (2014) | Websites that offer care over the Internet: is there an access quality tradeoff? | 2 |
| 25 | Deodhar, J (2002) | Telemedicine by email--experience in neonatal care at a primary care facility in rural India | 2 |
| 26 | Dias, V P et al. (2009) | Telenursing in primary health care: report of experience in southern Brazil | 2 |
| 27 | Dixon R F (2010) | Enhancing primary care through online communication | 7 |
| 28 | Dijksman, Ies et al. (2013) | eDiagnostics: a promising step towards primary mental health care | 2 |
| 29 | Eads, Michelle (2007) | Virtual office visits: a reachable and reimbursable innovation | 3 |
| 30 | Evans, L. (2001) | The use of e-mail by doctors in the West Midlands | 2 |
| 31 | Fashner, J & Drye, S T (2011) | Internet availability and interest in patients at a family medicine residency clinic | 2,4 |
| 32 | Fatehi, F. et al. (2014) | Design of a randomized, non-inferiority trial to evaluate the reliability of videoconferencing for e-consultation of diabetes | 3 |
| 33 | Fortney, J C et al. (2011) | A re-conceptualization of access for 21st century healthcare | 3 |
| 34 | Franklin VL et al. (2008) | Patients’ engagement With “Sweet Talk”–a text messaging support system for young people with diabetes | 2 |
| 35 | Gaster, B., et al. (2003). | Physicians' use of and attitudes toward electronic mail for patient communication. | 3 |
| 36 | Goyder C, Atherton H, Car M, Heneghan CJ, Car J. (2015) | Email for clinical communication between healthcare professionals. Cochrane Database Syst Rev. | 12 |
| 37 | Greenhalgh T et al. (2012) | The organising vision for telehealth and telecare: discourse analysis | 2, 10 |
| 38 | Greenhalgh T et al. (2013) | What matters to older people with assisted living needs? A phenomenological analysis of the use and non-use of telehealth and telecare | 2 |
| 39 | Greiver, M (2006) | Practice tips. E-mailing patients | 3 |
| 40 | Griffiths, F & Cave, J (2013) | Use of e-mail for consulting with patients in general practice | 3 |
| 41 | Gunning, E & Richards, E (2014) | Should patients be able to email their general practitioner? | 2 |
| 42 | Guy, R et al. (2012) | How effective are short message service reminders at increasing clinic attendance? A meta‐analysis and systematic review | 2 |
| 43 | Houston, T. K., et al. (2003) | Experiences of physicians who frequently use e-mail with patients | 3 |
| 44 | Katz, S. J., et al. (2003). | Effect of a triage-based E-mail system on clinic resource use and patient and physician satisfaction in primary care: a randomized controlled trial. | 3 |
| 45 | Kelaher, M. et al. (2006) | Improving access to medicines among clients of remote area Aboriginal and Torres Strait Islander Health Services | 2 |
| 46 | Kenny, C. & Steele, K. (2000) | E-mail consultations | 3 |
| 47 | Keely E et al. (2013) | Utilization, benefits, and impact of an e-consultation service across diverse specialties and primary care providers | 2,4 |
| 48 | King G (2007) | Adoption of telemedicine in Scottish remote and rural general practices: A qualitative study | 2,4 |
| 49 | Lester, W T (2004) | Facilitated lipid management using interactive e-mail: preliminary results of a randomized controlled trial | 2 |
| 50 | Liederman, E. M. and C. S. Morefield (2003). | Web messaging: a new tool for patient-physician communication. | 3 |
| 51 | Lim MS et al. (2008) | SMS STI: a review of the uses of mobile phone text messaging in sexual health | 2 |
| 52 | Lyngstad, M et al. (2014) | Home care nurses' experiences with using electronic messaging in their communication with general practitioners | 2 |
| 53 | Lyngstad, M et al. (2013) | Toward increased patient safety? Electronic communication of medication information between nurses in home health care and general practitioners | 2 |
| 54 | Markwick, L., K. McConnochie and N. Wood. (2015) | Expanding Telemedicine to Include Primary Care for the Urban Adult | 3 |
| 55 | Macfarlane, A et al. 2006) | A qualitative study of communication during joint teleconsultations at the primary-secondary care interface | 6 |
| 56 | May C (2003) | Health technology assessment in its local contacts: studies of telehealthcare | 2 |
| 57 | May C (2003) | Understanding the normalization of telemedicine services through qualitative evaluation | 2 |
| 58 | Moyer, C.A., et al. (2002). | Bridging the electronic divide: patient and provider perspectives on e-mail communication in primary care | 3 |
| 59 | Nieuwboer, C C (2015) | Single session email consultation for parents: An evaluation of its effect on empowerment | 2,9 |
| 60 | North F, Uthke LD, Tulledge-Scheitel SM. (2015) | Internal e-consultations in an integrated multispecialty practice. | 6 |
| 61 | Ohinmaa, A. et al. (2015) | The use of videoconferencing for mental health services in Finland | 2,5,10 |
| 62 | Olayiwola, J. N., D. Anderson, N. and I. Zlateva. (2016) | Electronic Consultations to Improve the Primary Care-Specialty Care Interface for Cardiology in the Medically Underserved | 6 |
| 63 | Oudshoorn N (2012) | How places matter: Telecare technologies and the changing spatial dimensions of healthcare | 2 |
| 64 | Rodgers A et al. (2005) | Do u smoke after txt? Results of a randomised trial of smoking cessation using mobile phone text messaging | 2,9 |
| 65 | Sands, D Z (2004) | Help for physicians contemplating use of e-mail with patients | 2 |
| 66 | Sciamanna, C.N., et al. (2007). | Patient access to U.S. physicians who conduct internet or e-mail consults. | 3 |
| 67 | Spencer, D C (2005) | Innovations in the practice of primary care: communicating with patients through E-mail | 3 |
| 68 | Steventon A (2012) | Effect of telehealth on use of secondary care and mortality: findings from the Whole System Demonstrator cluster randomised trial | 2 |
| 69 | Theodore, B. R., J. Whittington, C. and A. Z. Doorenbos. (2015) | Transaction cost analysis of in-clinic versus telehealth consultations for chronic pain: | 6, 2 |
| 70 | Tumolo, J. (2001) | You've got mail! E-mail in the 21st-century practice | 3 |
| 71 | Turner, T.H. et al. (2012) | A pilot trial of neuropsychological evaluations conducted via telemedicine in the Veterans Health Administration | 2,5,10 |
| 72 | Ulzen, T et al. (2012) | A pilot trial of neuropsychological evaluations conducted via telemedicine in the Veterans Health Administration | 2,5,10 |
| 73 | van Gurp, J., M. van Selm, and J. Hasselaar. (2016) | Teleconsultation for integrated palliative care at home | 6 |
| 74 | Viers et al (2015) | Efficiency, satisfaction and costs for remote video visits following radical prostatectomy: a randomised controlled trial | 6 |
| 75 | von Wangenheim, Aldo et al. (2012) | User satisfaction with asynchronous telemedicine: a study of users of Santa Catarina's system of telemedicine and telehealth | 2,4 |
| 76 | Wade et al (2010) | Wade, V. A., et al. (2010). "A systematic review of economic analyses of telehealth services using real time video communication." BMC Health Services Research 10: 233-233. | 5 |
| 77 | Wade et al 2015 | An evaluation of the benefits and challenges of video consulting between general practitioners and residential aged care facilities | 6 |
| 78 | Wallace et al (2002) | Design and performance of a multi-centre randomised controlled trial and economic evaluation of joint tele-consultations | 1 |
| 79 | Walters, B et al. (2006) | "Patient Portals" and "E-Visits" | 2,4 |
| 80 | White, C. B., et al. (2004). | A content analysis of e-mail communication between patients and their providers: patients get the message. | 3 |
| 81 | Zhang, Xiaojun et al. (2014) | Patients' adoption of the e-appointment scheduling service: A case study in primary healthcare | 2,4 |

| **Reasons** | |
| --- | --- |
| 1 | Does not have full citation details/ unable to source (such as journal) or is a protocol. |
| 2. | Is not centrally focused on *remote/ virtual / e-consultation* in primary care settings. |
| 3.  4.  5.  6.  7.  8.  9.  10.  11.  12 | The paper is not primary research; or is already included in a systematic review/ meta-analysis or an economic study already included in this review.  The study does not use a specific e-consultation tool (such as email, visual / video technologies i.e. skype/ other  The paper does not centrally focus on primary care.  E-consultations must involve the patient. As such, provider-to-provider interactions are excluded from this review. All included studies must involve the patient in the e-consultation with their primary care provider.  The experimental evidence fails to provide specific outcomes measures.  The paper relates to the use of the telephone in general practice.  The paper relates to online / e-Health health promotion / education tools.  The paper relates to telehealth/ telemonitoring of chronic conditions.  Focuses on systems / services administration tools that are not linked to the e- consultation process (e.g. invitations to patients to participate in research projects). Or do not form part of a virtual consultation which is directly relevant to direct patient care.  Systematic reviews with no new updates |
