# Supplementary material for: Electronic Consultation in Primary Care Between Providers and Patients: Systematic Review
Source: JMIR Med Inform. 2019 Dec 3;7(4):e13042. doi: 10.2196/13042 (PMC6918214; doi:10.2196/13042)
Supplement: Multimedia Appendix 3 [file medinform_v7i4e13042_app3.docx]

**Multimedia Appendix 3 - MMAT Scores**

| **Reference** | **Research Question** | | **Methodology** | **MMAT %** |
| --- | --- | --- | --- | --- |
|  | Clear RQ? | Objectives? |  |  |
| Adamson (2010 ) | N | Y | Quantitative Descriptive | 50 |
| Alder (2006) | Y | Y | Quantitative Descriptive | 75 |
| Albert (2011) | Y | Y | Mixed Methods | 50 |
| Angstman (2009) | Y | Y | Quantitative Non-RCT | 25 |
| Atherton (2013) | Y | Y | Qualitative | 100 |
| Atherton (2012) | Y | Y | Review | - |
| Baldwin (2002) | N | Y | Qualitative | 50 |
| Bishop (2013) | Y | Y | Qualitative | 75 |
| Brown-Connolly (2002) | Y | Y | Quantitative Descriptive | 75 |
| Brunett (2015) | Y | Y | Quantitative Descriptive | 75 |
| Caffery (2010) | Y | Y | Review | - |
| Cooper (2012) | N | Y | Quantitative Descriptive | 75 |
| Couchman (2005) | N | Y | Quantitative Descriptive | 100 |
| Davis (2013) | Y | Y | Qualitative | 100 |
| Denberg (2007) | Y | Y | Quantitative Descriptive | 50 |
| Delbanco (2012) | Y | Y | Quantitative Non-RCT | 100 |
| Dixon (2008) | Y | Y | Quantitative Non-RCT | 0 |
| Elliott (2007) | Y | Y | Quantitative Descriptive | 75 |
| Granlund (2003) | Y | N | Quantitative Non-RCT | 25 |
| Grayston(2010) | Y | Y | Quantitative Descriptive | 75 |
| Greenhalgh(2010) | N | Y | Mixed Methods | 100 |
| Hanna (2011) | Y | Y | Mixed Methods | 100 |
| Hanna (2012) | Y | Y | Qualitative | 75 |
| Hansen (2014) | Y | Y | Qualitative | 50 |
| Hanson (2009) | Y | Y | Quantitative Non-RCT | 25 |
| Harrison (2006) | Y | Y | Qualitative | 75 |
| Hickson (2014) | Y | Y | ReviewReview | - |
| Jacklin (2003) | Y | Y | Quantitative RCT | 50 |
| Jiwa (2013) | Y | Y | Quantitative Descriptive | 75 |
| Kittler (2004) | Y | Y | Quantitative Descriptive | 50 |
| Liddy (2013b) | N | Y | Mixed Methods | 75 |
| Mehrotra (2013) | Y | Y | Quantitative Descriptive | 75 |
| Mirsky (2016) | Y | N | Qualitative | 50 |
| Neville (2004b) | Y | Y | Mixed Methods | 75 |
| Nilsson (2009) | Y | Y | Quantitative Non-RCT | 100 |
| North (2013) | Y | Y | Quantitative Descriptive | 100 |
| North (2014) | Y | Y | Quantitative Descriptive | 75 |
| Padman (2010) | Y | N | Mixed Methods | 25 |
| Pagliari (2005) | Y | Y | Quantitative Descriptive | 75 |
| Palen (2012) | Y | Y | Quantitative Non-RCT | 75 |
| Polinski (2015) | Y | Y | Quantitative Descriptive | 50 |
| Popeski (2015) | Y | Y | Mixed Methods | 75 |
| Ralston (2009) | Y | Y | Quantitative Non-RCT | 75 |
| Richards (2005) | N | Y | Quantitative Descriptive | 100 |
| Riippa (2015) | Y | Y | Quantitative Non-RCT | 50 |
| Rohrer (2013) | Y | Y | Quantitative Descriptive | 75 |
| Roter (2008) | Y | Y | Qualitative | 50 |
| Schattner (2008) | Y | Y | Qualitative | 50 |
| Sevean (2008) | Y | Y | Qualitative | 75 |
| Shimada(2013) | N | Y | Quantitative Non-RCT | 50 |
| Torppa (2006) | Y | Y | Qualitative Qualitative | 100 |
| Umefjord (2004) | Y | Y | Quantitative Descriptive | 25 |
| Umefjord (2006) | Y | N | Quantitative Descriptive | 50 |
| Wakefield (2012) | Y | N | Quantitative Descriptive | 50 |
| Wallace (2004) | Y | Y | Quantitative RCT | 100 |
| Ye (2010) | Y | Y | Review | - |
| Zanaboni (2009) | Y | Y | Quantitative Descriptive | 25 |

**MMAT Key:**

Y: Yes;

N: No;

C: Can’t tell

R: Review

**Study Characteristics Table**

| **Study Type** | | **Reference/ Code** |
| --- | --- | --- |
| **Qualitative** | | |
| **1.** | Case studies/management/ review of medical records/ messaging analysis (**n=4)** | Baldwin L, Clarke M & Jones R. (2002) (UK)  Neville, R. et al. (2004b) (UK)  Mirsky JB,et al . (2016) (USA)  Roter, D. et al. (2008) (USA) |
|  | Interviews/ Focus groups **(n=9)** | Atherton, H. et al. (2013) (UK)  Hanna, L. et al. (2012) (UK)  Harrison, R. et al. (2006) (UK)  Bishop T. (2013) (USA)  Davis, M. et al. (2013) (USA)  Schattner P, Matthews M & Pinksier N. (2008) (Australia)  Sevean, P. et al. (2008) (Canada)  Hansen, CS. et al. (2014) (Denmark)  Torppa, M. et al. (2006) (Finland) |
| **Quantitative RCT** | | |
| **2.** | Randomised Control Trials (RCT) **(n=2)** | Jacklin, PB. et al. (2003) (UK)  Wallace, P. (2004) (UK) |
| **Quantitative, non-randomised** | | |
| **3.** | Quasi-experimental (trial and survey) (open and controlled studies, sequential control design) **(n=3)** | Delbanco, T. et al. (2012) (USA)  Granlund, H. et al (2003) (Finland)  Riippa, I. e al (2015) (Finland) |
|  | Cohort studies (retrospective, prospective and longitudinal) **(n=4)** | Hanson D, Calhoun J & Smith D. (2009) (USA)  Ralston, JD. et al. (2009) (USA)  Shimada, S. et al. (2013) (USA)  Nilsson, M. et al. (2009) (Sweden) |
|  | Cross-sectional/ analysis including various modes of delivery; telephone, online). Retrospective case control studies. Sequential control design. **(n=3)** | Angstman, KB. et al. (2009) (USA)  Dixon, RF & Stahl, JE. (2008) (USA)  Palen, TE. et al. (2012) (USA) |
| **Quantitative descriptive studies** | | |
| **4** | Quantitative, descriptive study (pilot study reporting service use and frequencies, survey/ analysis, descriptive vignette study, quantitative analysis of medical records/ secure messaging, retrospective survey) **(n=22)** | Grayston, J. et al. (2010) (UK)  Pagliari, C. et al. (2005) (UK)  Richards, H. et al. (2005) (UK)  Umefjord, G. et al. (2004) (Sweden)  Umefjord, G. et al. (2006) (Sweden)  Zanaboni, P. et al. (2009) (Italy)  Jiwa, M & Meng, X. (2013) (Australia)  Adamson, SC. et al. (2010 ) (USA)  Alder, KG. et al. (2006) (USA)  Brown-Connolly, NE. (2002) (USA)  Brunett, PH et al. (2015) (USA)  Cooper, CP. et al. (2012) (USA)  Couchman, GR. et al. (2005) (USA)  Denberg T, Ross S, & Steiner J. (2007) (USA)  Elliott J, Chapman J, & Clark D. (2007) (USA)  Kittler, AF. et al. (2004) (USA)  Mehrotra, A. et al. (2013) (USA)  North, F. et al. (2013) (USA)  North, F. et al. (2014) (USA)  Polinski JM et al. (2015) (USA)  Rohrer, JE. (2013) (USA)  Wakefield, DS. et al. (2012) (USA) |
| **Mixed Methods** | | |
| **5.** | Mixed Methods (interviews, focus groups and quantitative analysis of based system utilisation data/ survey data)  **(n=6)** | Greenhalgh, T. et al. (2010) (UK)  Hanna L, May, C & Fairhurst K. (2011) (UK)  Liddy, C. et al. (2013b) (Canada)  Popeski, N et al. (2015) (Canada)  Albert, SM. et al. (2011) (USA)  Padman, R. et al. (2010) (USA) |
| **Reviews** | | |
|  | Interpretative/ Literature Reviews  **(n=4)** | Atherton, H. et al. (2012) (UK)  Hickson, R. et al. (2014) (USA)  Ye, J. et al. (2010) (USA)  Caffery LJ.& Smith AC. (2010) (Australia) |
