# Supplementary material for: Electronic Consultation in Primary Care Between Providers and Patients: Systematic Review
Source: JMIR Med Inform. 2019 Dec 3;7(4):e13042. doi: 10.2196/13042 (PMC6918214; doi:10.2196/13042)
Supplement: Multimedia Appendix 4 [file medinform_v7i4e13042_app4.docx]

**Multimedia Appendix 4 - Evidence Table**

| **Author, Year, Country**  **Aim of Study**  **E-consultation Type**  **Asynchronous, synchronous, mixed** | **Study Design, Population and Setting & Study Dates**  **Benefit to: Patients, clinicians, organisational/ technical, mixed** | **Outcome Measures, Comparator groups**  **and Analysis Methods** | **Results/ Findings** |
| --- | --- | --- | --- |
| Adamson SC et al, 2010.  USA  To learn about the potential for e-consultations – including structured online histories - in preparation for construction of an online patient portal to be used across the institution.  Asynchronous | A pilot study to learn about the possibility of using e-consultations in the Dept. of Family Medicine at Mayo Clinic, Rochester, USA.  The clinic averages n=56 clinicians across 4 clinics.  Data was collected over a 2 year period Nov 2007 – Oct 2009.  Scale: Local  Benefit to: patients, professionals/clinicians and organisation | No/No  No detailed explanation as to how data was analysed. | Most e-consultations occurred during working hours, primarily by females and involved 294 different conditions. An e-consultation cost $35 US.  Estimate of reduction in office visits of 999 (40%).  Use of structured history taking provided an organised history, with all medications, allergies, vital signs, and eliminated need for the physician to ask for further information.  Family registrations for e-consultations meant patients did not need to leave work to bring dependents to the office. |
| Adler KG et al. (2006)  USA  To evaluate whether current patients are ready and willing to pay for online services in a fairly typical family medicine practice.  Asynchronous | Anonymously survey (one month).  Targeted 346 Patients out of 2380 active patients (14.5%).  Response rate was 95.1% (329 of 346).  Primary care office -part of a family medicine office (3 physicians and 1 nurse practitioner), with 2380 active patients.  Scale: Urban local  Benefit to: patients and organisations  (mixed) | No/No  Fisher exact test | Those aged 18-29 year-olds had the highest access (97%, n=28/29 ) and patients 70 years and older had the least access (56%, (n=40/72), (P<.001).  Students (92%, n=24/26) and the employed (87%, n=123/ 142) has greater access.  Patients that were retired (66%, n=81/123) and the disabled (42%, n=10/24) had the worse access (P<.001).  Of those with internet access (n=248), 60.1% (n=149) were willing to pay US $10 or more per year; and 31% (n=46) up to US$50 or more per year. Willingness to pay did not significantly differ by age (P=.06).  The three most important services to patients with internet access (n=248) were: emailing physician (34%), viewing their record online (22%) and medication refills (11%) (P<.001) |
| Albert SM et al. (2011)  USA  To investigate experience and outcome (following 7 days) of the first 156 e-consultation users in University of Pittsburgh Medicine Centre (UPMC).  Asynchronous | Mixed= Telephone Survey / medical record review and interviews.  First n=121 users of HealthTrak e-consultation system (77.6% participation, n=156 users in total).  Large (7,000 patients) family medical practice with multiple sites - University of Pittsburgh Medicine Centre (UPMC).  Scale: Local  Equivocal results to both patients and clinicians. | No/No  Descriptive statistics, chi-square test, and t-tests for differences in mean scores. | 40.5% (n=70) participates stated "convenience" was their primary reason for e-consultation.  17.6 % (n=27) perceived their symptoms were for minor complaints and did not need a face-to-face visit.  11.8% (n=18) patient's reported that physicians were not available for face-to-face consultation at that time.  Over 90% of pts reported their health concerns were addressed and most did not need to return for an in-person visit. |
| Angstman KB et al. (2009)  USA  Evaluation of the impact of e-consultations (EC) on return visits for family medicine patients.  Asynchronous | Two group comparison EC requested (n=228) and face to face referrals (n=500) by reviewing electronic medical records.  Clinic (not specified) Practice size not specified.  Scale: Single practice study  Benefit to: patients and specialists. Disbenefit to GPs. | Yes/ Two-group comparison  Univariate tests  Chi square test  Means test  Multiple logistic regression | From the total 728 cases, 500 (68.7%) were seen by specialists, and 228 (31.3%) were signposted for an EC.  The EC group were slightly younger (mean age) (45.9 vs 50.3; P <0.01).  There was a high rate of return visit within 2 weeks for any reason (almost 40%, n=87), in contrast to 27.6% (n=63) of face-to-face visits (P <0 .01).  The only significant predictor of return visits for any reason was linked to patients receiving EC (OR, 1.88; CI, 1.33-2.66; P <0.01); patients with higher levels of comorbidities (OR, 1.78; CI, 1.15-2.74; P < 0.01), or patients that were older (OR, 1.01; CI, 100-1.02; P =0.03). |
| Atherton H et al (2013)  UK  To understand the use of email consultation in general practice by investigating the experiences of existing users and views of the experts.  Asynchronous | Semi-structured interviews.  Purposive sampling  N=30 (14 patients, 10 GPs and 6 experts)  General practice & community settings in London boroughs  Scale: Local  Benefit to: Equivocal results to both patients and GPs. | Yes / Thematic analysis using Atlas.Ti (v6) | Four themes emerged. E-consults were *initiated* by logistic or practical issues with the popularity of email being part of initiating its use. Once established; contact was largely initiated by the patients.  *Motivators* for ongoing use were convenience for GPs and patients and were seen as an alternative route to care for both parties.  *Impact:* GPs raised concerns with regard to potential rise in workload (GPs) and safety in terms of lack of guidance about the ‘rules of engagement’ in email consultations. Lack of guidance provoked uncertainty.  *Management:* Patients and GPs both attempted to alleviate the effects of email on workload, to improve safety and introduce rules of engagement. |
| Atherton, H et al. (2012)  UK  To assess the use email to communicate between patient and health care professional focusing on various outcomes; including patient outcomes, health care service performance, efficiency and acceptability.  Asynchronous | Cochrane Systematic Review  Scale=International  Benefit to: Equivocal results to patients, GPs/clinicians. | Multiple professional, patient and service outcomes. Safety.  Reported effect size (mean difference) (MD), Odds Ratio (OR) and Rate Ratio (RR) and mean values.  No meta-analysis of data could be performed.  Risk of Bias assessment. | The included nine trials were assessed to be limited, with variable results, missing data and were assessed to be at Risk of Bias. As such the evidence was not able to adequately assess whether the effect of email for communication between patients/caregivers and healthcare professionals was effective.  It was not possible to assess whether email had any effect on patient/caregiver outcomes; when compared to standard communication methods. Some evidence indicated that email communication may lead to a rise of email and telephone calls for healthcare groups.  Evidence relating to adverse events was unclear with three studies reporting some type of adverse event but limited knowledge about the impact on patient health outcomes or quality of care. |
| Baldwin L, Clarke M & Jones R. (2002)  UK & Greece  To describe and discuss the use of a virtual consultation system (AIDMAN) in various settings focusing on diagnosis and case management.  Asynchronous | A descriptive analysis of 40 video consultations.  A case management study.  Settings: various including Greece (remote islands) and the UK.  Four hospitals in Greece, one in Athens and three in remote health care centres in the islands of Corfu, Mykonos and Mytilini.  AIDMAN was installed in a primary care clinic/general practice in Chorleywood UK. Scale=International  Benefit to: patients and health care professionals (mixed) | Yes/ Patients and health care professionals/  No detailed explanation as to how data was analysed.  Video recordings of virtual consults with a range of patients in one UK GP practice over a 2 year period. | A typical tele-consultation was assessed to be composed of 15% (n=6) investigation, 15% (n=6) management, 10% (n=4) introduction to the problem, 10% (n=4) introduction to the patient, 50% (n=20) history.  Patients were provided with opportunities to learn more about many aspects of their health and how better to manage their conditions.  AIDman was reported to provide specific advantages over traditional forms of consultation; mainly regarding the ability to manage patients cases more swiftly where issues are resolved at end of the tele-session rather than over time (weeks or months). |
| Bishop T. (2013)  USA  A qualitative study to explore how can primary care practices use electronic communication to manage clinical issues that are traditionally managed during office visits?  Asynchronous | A qualitative semi-structured interview study.  Six medical groups (5 of which were defined as large (between 115-500 physicians).  Scale=National  Benefit to: patients and health care professionals (mixed) | Interviews coded using Atlas.ti qualitative analysis software (v6.2) using the constant comparative method. | More advantages than disadvantages were reported by interviewees who stated that electronic communication improved access to care for patients, saved patients time and improved patient satisfaction.  Physicians reported electronic communication as being efficient for them with each email or secure messages taking little time.  The biggest disadvantage reported was added work lengthening the working day. With the increase of electronic communications, attempts were made to reduce the number of office visits made with little success.  A "key barrier" was the traditional fee-for-service payment model. |
| Brown-Connolly NE (2002)  USA (California)  To assess patient satisfaction with using telemedicine in California; compared to face-to-face care.  Mixed | Questionnaire survey.  N=793 / 1301 tele-consultations (response rate was 61%) adult/child patients.  Setting= mixed (24 primary care sites in 18 Californian counties).  Data collected over a 20 month period (Sept 1999- April 2001).  Scale=National  Benefit to: both patients and clinicians. | No/No  (1) Patients satisfaction with the services (2) number of consultations (3) and sources of payment analysis conducted using descriptive statistics. | 91% (n=722, mean 4.6) of participant indicated telemedicine made it easier for patients to receive speciality care.  In general, 87% (n=690, mean=4.5) of participant was satisfied with telemedicine. 90% (n=714, mean=4.6) of participant showed the willingness to continue receiving services.  61% (n=484, mean=2.3) of participant indicated they would not receive better care than in person.  N= 741 patients submitted travel information, the results indicated an average decrease in distance of 170 KM (one way), and time-saving of 130 mins when telemedicine caused. The average cost of travel to a speciality appointment was $83.16 (n=310). |
| Brunett PH et al. (2015)  USA (Portland)  To determine the feasibility of voice and video internet technology use as compared to in-person urgent clinic visits.  Mixed | Retrospective chart review.  N=456 patients seen via internet-based technology.  Setting = 24 clinics in the Northwest United States.  Data collected in the pilot stage: Oct 11th 2011- June 30^th^ 2012.  Scale= Regional  Benefit to: both patients and clinicians | No/No  Frequency tables and descriptive statistics.  Fisher’s exact test was used to calculate  p-values.  Analysis conducted using R (statistical computing) v. 3.0.2 | Female patients were the largest number within the cohort 69.8% (n=318). The largest female subgroup were those aged 30 to 39 years (31.7%, n=145), followed by females aged 40 to 49 (19.5%, n=89).  The most common complaints for online visits were sinusitis (23.2%, n=111), female UTIs (20.0%, n=96), and rashes (12.5%, n=60), all considered to be routine, and non-urgent conditions.  No online or in-person visit resulted in an emergency referral, hospitalization or need for a paramedic.  Real-time internet visits could be successfully completed with minimal technical difficulties. Out of the 478 Internet visits, only three visits (<1%) had to be aborted because of technology problems.  They conclude that real-time internet visits are feasible, safe and potentially beneficial in improving convenience, and access to urgent and primary care. |
| Caffery, Liam J.  Smith, Anthony C. (2010)  Australia  To review 185 peer-reviewed studies focusing on email-based health service delivery.  Asynchronous | Structured literature review (n=185 included studies)    Scale=International  Benefit to: equivocal results to patients, clinicians and organisations. | No/No  Structured analysis | Email-based telemedicine does not replace face-to-face consultations in the majority of services but augments them with complementary consultations.  Workload and reimbursement are the two main reasons that clinicians were reluctant to use email consultation.  There is a clear need to encrypt emails to secure patient confidentiality; the need may be legislative or ethical.  Email consultation changes the relationship between health providers and patients.  Email offers the opportunity for reluctant patients to use a medical service. |
| Cooper, Crystale Purvis et al. (2012)  USA  To investigated the demographic and practice-related characteristics of US physicians using internet-based communication technologies such as websites, and portable devices to communicate with patients.  Asynchronous | Survey/ Sample from the DocStyles (2009) survey drawn from the Epocrates Honours Panel including 156,000 US Physicians.  Data was analysed from 1750 physicians in 5 specialties (family medicine, n=609: internal medicine n=391: Paediatrics, n=250: Obstetrics/Gynaecology, n=250: Dermatology, n=250)  Scale= National  Benefit to: patients. | No/No  Univariate logistics regression and multivariate, forward-stepwise logistic regression using SPSS (v19.0) | DocStyles is an annual web-based survey which investigates the attitudes and clinical practices of US physicians and allied health professionals.  Most family practice patients have e-mail access; such access among these patients varies among practices.  Family practice patients especially want to use E-mail to refill prescriptions.  Patients have high expectations that email services will be very prompt. |
| Couchman, Glen R.  et al. (2005)  USA  To assess patients willingness to access test results, prescription requests (and other services) and assess their expectations regarding the timeliness of use.  Asynchronous | Cross-sectional survey.  Population= 2260/ 186,000; Adults - Patients; no dates provided.  N=3652 patients were approached. N=2314 (82.1%) completed surveys.  Participates under 18 years old and those that could not speak English were excluded.  Mixed settings (Scott & White HealthCare Systems, a multi-speciality, university-affiliated group practice, in central Texas, USA).  Practice No=19; Practice size= large.  Scale=Regional  Equivocal results. | No/No  Chi-square test/ Fisher's exact test.  Nonparametric Kruskal-Wallis analysis of variance for ordinary (Likert-style data) | Over half of pts (58.3%, n=1349) had e-mail access, which is much lower than in the UK (84.1%). Only 5.8% (n=134) had used email to communicate with their doctor.  Patients were only willing to use email for specific types of communication. For example:   - to request prescription refills (83%; n=1921, mean=4.02); - to communicate directly with their physician (82%, n=1897; mean=3.88); - for non-urgent consultation (82%, n=1897; mean=3.86); - to obtain routine laboratory (82%, n=1897; mean=3.85); - to book an appointment (74%, n=1712; mean=3.50).   Patients expected timely replies to their email.  Patients were willing to use email to access laboratory results for routine issues such as cholesterol (85%, n=1967; mean = 3.90, or blood sugar (84%, n=1944; mean = 3.86) but were less inclined to use it for other, more serious, issues such as Brain CT scan (59%, n=1365 ; mean = 3.05). |
| Davis M, et al. (2013)  USA  To explore rural primary care clinicians’ interest and resources necessary to incorporates Remote Monitoring Technologies (RMTs) into routine practice.  Synchronous | N=15 in-depth interviews with rural primary care clinicians from November 2011 to April 2012.  Purposive sample based on geographic diversity (large rural, small rural and isolated).  The Oregon Rural Practice-based Research Network was the source of participant population (n=131 members across 41 practices)  Scale=Regional.  Equivocal results to both clinicians and patients. | Yes/Cross case comparison.  Thematic analysis and cross-case comparative analysis | Clinicians interviewed were interested in RMTs which was closely aligned to the goals of primary care practice, capable of implementation with least difficulty.  No consistent differences were discernible in the cross-case comparison in perceptions towards RMTs by clinical training age, gender, practice size or other anticipated characteristics.  Clinicians noted that relevant technologies could be used for different age groups. |
| Denberg T, Ross S, Steiner J. (2007)  USA  To determine patient acceptance of a preventive care model employing outreach by non-physician experts outside of clinic visits. Including wiliness to communicate with experts by phone, email or the internet.  Mixed | Survey.  N=354 (August-September 2005)  Setting= a general medicine clinic associated with the University of Colorado.  Scale=One single clinical practice  Equivocal results. | Non-parametric methods, two-sided Chi-square tests using Stata 7.0 | More than 70% (n=248) of patients were open to receiving non-PCP centred methods of receiving preventive services.  Socioeconomically disadvantaged patients and those with poorer self-rated health were less likely to express an interest in the concept |
| Delbanco, Tom  et al. (2012)  USA  To evaluate the effect of facilitating patients access to their visit notes through a secure internet portal and electronic messaging and the impact of this access on the doctors work lives.  Mixed | Quasi-experimental trial and survey.  N=105 physicians and N=13,564 adult patients (trial); 41% of 13,564 (completed survey) N=5,561  2011-no end date  Setting= 3 mixed primary care practices (city x2 &rural x1, in Massachusetts, Pennsylvania and Washington, USA)  Scale=Regional  Benefit to: patients. | No/No  Descriptive statistics  Cochran-Mental-Haenszel.  Regression models using SAS software (v9.3) | Patients were enthusiastic about open access to electronic messaging.  A large number of patients opened some or all of their notes, had minimal concerns and almost all believed that open notes would affect their decision-making skills.  The benefits to patients were realised with far less impact on the work-life of doctors.  Most of the doctors think opening their notes to patients online is able to:   - strengthen relationships with some of their patients, - some patients seemed more activated and empowered; - improve patients' satisfaction, patient safety, the ability to reinforce the office visit and the opportunity for patient education; - increase in efficiency. |
| Dixon, Ronald F.  Stahl, James E. (2008)  USA  To investigate the feasibility, effectiveness, and acceptability of a patient-physician real-time encounter using video conferencing technology (a virtual visit) compared to a face-to-face office visit.  Synchronous | A sequential (pilot) control design comprising of a virtual visit with a general practitioner and the completion of a pre and post evaluative questionnaire for both clinician and patient.  N=30 adults (18-85 years) were recruited from a single primary care practice.  Subjects were recruited sequentially regardless of a complaint by one single clinician.  Scale= Single Local  Equivocal results to patients and clinicians. | Yes/ To compare the doctor's ability to diagnose in both real-time and virtually; to compare doctors ability to provide therapy using both methods, and to examine both patient and their doctor's sense of satisfaction with both modalities.  Data were analysed using two-tailed t-tests and analysis of variance. | Patients and physician were surveyed after each visit type with regard to the quality of the history, quality of the examination, and satisfaction with the experience. Patients significantly preferred the in-person visit (4.7 of 5) but were very satisfied with the virtual visit as well (4.1 of 5) (p < 0.0001).  Quality of the physical examination varied as physical examination effectiveness was significantly worse in the virtual visit modality (2.3 versus 4.9 for the face-to-face visit, p < 0.0001), but history and therapeutic effectiveness were not significantly different.  Patient and their doctors both felt comfortable with using the technology (patients 4.1, doctors 4.3). |
| Elliott J, Chapman J, & Clark D. (2007)  USA  To determine the feasibility of using video conferencing to conduct pain clinic follow-up visits.  Synchronous | Prospective, descriptive, quantitative exploratory design.  Questionnaire.  Pain clinic patients (n= 36).  Clinic staff n= 1 physician, n=1 nurse specialist and rotating  Psychology interns.  Data collected over 29 months  Setting: The Veterans Affairs Health Care System in Palo Alto, California  Scale= Single Local  Benefit to: patients and clinicians. | Yes/ Use of videoconferencing for stable pain clinic users is useable and satisfactory for both patients and staff./ No  Descriptive statistics | Patients overall satisfaction was (76%, n=27) and rated VC as either excellent or very good.  The average one-way distance saved per patient was 65 miles (24-89 miles range). The average time saved was 126 minutes one way (range 80-235 minutes).  Staff reported satisfaction to be good/very good (88%, n=117/133); but the ability to communicate (11%; 11/133) indicating face-to-face contact to be preferable.  90% (n=120) staff responses indicated videoconferencing to be superior to telephone calls, and 32% (42/133) suggesting staff could identify new problems during the Telepain visit.  Negative responses to use were a lack of familiarity with the equipment. |
| Granlund H, Thoden C-J, Carlson C, Harno K (2003)  Finland  The comparison of immediate and six-month outcomes of real-time video conferencing (RTV) and face-to-face (FTF) consultations.  Synchronous | An open controlled study with a questionnaire (open and non-randomised).  N=46 (23 in RTV arm, 25 in FTF)  29 in the 6 months follow up.  1 year recruitment period.  N=2 health centres in the suburbs of Helsinki  Practice size: not specified.  Scale=Local  Equivocal results to both patients and clinicians. | Yes/Yes, Face-to-face and real-time video conferencing.  Student's t-test for between-group comparisons  Paired t-test for within-group comparisons  The Wilcoxon rank-sum test with one-tailed interpretation  Dichotomous variables (proportions) were tested using Chi-square tests or Fishers' exact test. | Patients were significantly more satisfied with RTV rather than FTF groups (median LAS score being 9.6 in the RTV group and 9.0 in the FTF group) (p=0.03).  Consultant satisfaction was linked to problems with screens freezing (16%, n=5) or sporadic (41%, n=12) image.  Generally, GPs were more confident with FTF, but history taking with RTV was more successful.  There were no significant differences in management plans but more treatment suggestions were given to GPs with RTV consultations. |
| Grayston, J. et al. (2010)  UK  To assess patients attitudes towards three types of technology; a messaging service (SMS), webpages and email for the delivery of laboratory results.  Asynchronous | A structured interview with n=200 patients aged between 18 and 87 years.  Patients attending a doctor’s, nurse or phlebotomist appointment.  Practice size: Two general practices located in Lothian, Scotland.  Scale= Local  Benefit to: clinicians  Disbenefit to: patients | Satisfaction measured on a 5 point Likert scale.  Data analysed by age, sex and educational attainment using *χ*^2^ test  Sensitivity analysis performed. | Most patients telephoned the local practice for their test results (n=75, 38.3%).  Only 11 (4.2%) of responses indicated any of the preferred 3 new technologies being considered.  E-mail accounted for n=8 (3%), whilst SMS (0.8%) and webpage (0.4%) were scored lower.  Of those that had access to email; just over half (n=75, 53.3%) thought it was favourable to receive test results in this way. There was no significant difference in either age or gender.  Reasons for favourable attitudes to email included speed and efficiency (44.0%, n=88), and convenience (30.7%, n=61). The main concerns were related to information security, confidentiality (33.33%, n=7) and a sense that email was impersonal (16.0%, n=32). |
| Greenhalgh, T. et al. (2010)  UK  To evaluate the policy-making process, an implementation by NHS organisations, and patients and carers experiences of efforts to introduce an internet-accessible personal electronic health record (HealthSpace) in a public sector healthcare system.  Asynchronous | A mixed-method, multilevel case study including observational field notes, interviews and review of national registration statistics,  N=56 patients and carers. N=160 interviews of staff in national and local organisations.  Data collected between 2007 and Oct 2010  Scale=national  Disbenefit to patients.  Equivocal results to patients, and clinicians | Qualitative empirical data analysis via provisional analytical categories using a constant comparative method.  Narrative was used to blend qualitative and quantitative findings. | N=172950 people opened a basic HealthSpace account and 0.13% (n=22) opened an advanced account.  HealthSpace was perceived to be neither useful nor easy to use and functionality aligned poorly with their expectations and self-management practices.  Users of email style messaging were positive about its benefits but there were only 3 early adopter clinicians, fewer than 100 out of 3000 expressed interest.  Over the 3 year evaluation, period policymakers hoped that HealthSpace would lead to empowered patients, personalised care, lower NHS costs; lead to better quality data and improved health literacy. These were not realised. |
| Hanna L, May, C & Fairhurst K. (2011)  UK (Scotland)  To explore practice managers' views of remote consultations and communication technologies in the delivery of primary care.  Mixed | Mixed=postal survey and in-depth interviews with adult patients, carers and representatives.  N=600 (survey)  N=20 (interviews)  Population= not specified (all practice managers in Scotland);  Practice No= 1026 Practice size= mixed - a range of practice list sizes, geographical locations and practice area deprivation indices.  Scale=national  Benefit to: both patients and clinicians. | Qualitative data analysis of interviews using a constant comparative method to identify themes. Analysis facilitated by use of QSR NVIVO | Practice managers play a key role in any service redesign to introduce remote consultation/communication technologies in primary care.  Practice managers' views on the appropriateness of these technologies are influenced by a mix of contextual and practice characteristics, such as locality, practice size, practice team ICT capacity and the nature of practice population.  Their major concerns are medico-legal and perceived lack of patient demand. Practice managers stressed clear external guidance is needed to ensure ongoing quality and safety. |
| Hanna, L. et al. (2012)  UK (Scotland)  To explore GP's perceptions of the potential place of new non-face-to-face consultation technologies in the routine delivery of primary care.  Asynchronous | Semi-structured interview with N=20, Adults - Carers/representatives (practice managers); no dates  Population= not specified (all practice managers in Scotland);  Practice No= 1026 (practices)  Practice size= other (mixed - a range of practice list sizes, geographical locations and practice area deprivation indices.  Scale=national  Benefit to: clinicians | Interview data analysed using a constant comparative method to identify and develop categories. Analysis facilitated by use of QSR NVIVO | Many GPs were conditionally willing to consider using new technologies in the future, especially for administrative or less complete tasks to maximise practice efficiency and patient convenience.  GPs barriers to the use of e-consultation technologies were; reliability of technology, lack of perceived patient demand, inequalities in access to primary medical care, the increase of workload, medico-legal concerns and effect on doctor-patient relationships.  Most GPs consider an adequate resource, support and further training from their health board and clear-legal guidance to help guide them in communicating with patients. |
| Hansen, C S  et al. (2014)  Denmark  The study investigates the ways in which patients and general practitioners communicate with each other by e-mail, identifying the factors influencing this means of communication and puts into perspective the potential of e-mail consultations inpatient treatment.  Asynchronous | A qualitative study using individual interviews and 4 focus groups.  N=5 GPs. N=14, patients  Patient recruitment was via an internal mailing list at Roskilde University and a general practice clinic in the capital region of Denmark.  Scale=regional  Equivocal results to both patients and clinicians. | GP/ Patient data comparison.  Empirical data was analysed using a social constructivist and practice theoretical approach. | GPs and patients have different perceptions of the purposes for which e-mail consultations are suitable.  Patient use of email gave patients the chance to formulate their agenda in their own time, to review the GP response and to get actively involved in their treatment. E-mail could be used for information/ image sharing and for signposting patients to articles and websites.  Patients found that when GPs responded that a question was not appropriate for e-mail, the patient felt rejected.  Patients did not always consider the urgency of their e-mail query before making it and preferred quick responses from their GPs, preferably the same day. |
| Hanson D, Calhoun J, Smith D. (2009)  USA  A longitudinal study comparing pre and post-real-time video conferencing facilitates focusing on changing attitudes and direction of attitude change between first-time and experienced video conferencing users.  Synchronous | A longitudinal pre/post questionnaire study of healthcare providers attitudes towards real-time video conferencing.  Population: n=50 Physicians from the Medical College of Georgia Medical Centre and (n=37) healthcare providers from mid-Nebraska Telemedicine Network.  A total of n=87 providers completed the questionnaire prior to and after each telemedicine encounter.  Practice size= Majority were group practices (n=25/56 sites – 1^st^ time, n=12/31 experienced).  January 1995 to March 1997.  Scale= Regional  Disbenefit to patients (over time). | Chi-square test. Fisher's exact test.  Impact of the system on productivity and ability to prescribe treatment. | Most of the study population across both settings were aged between 31-49 years (75%, n=42/56 - 1st-time users and n=21/31 experienced users). More males (68%, n=39/56 1^st^ time, n=20/31 experienced) than females (30%, n=17/56 1^st^ time, 35%, n=11/31 - experienced) took part.  Only n=18 providers who did change their views after the telemedicine encounter was more positive among those who used the system for the first time; as compared to those with experience.  In contrast, experienced users became more negative (p=0.025) suggesting that experience of systems result in more positive attitudes that may not last to subsequent interactions with the technology. As such there are differences in the direction of change between the attitude changes of these groups.  1st-time users are somewhat cautious in their attitudes – it may be important to generate more positive views by the support of leaders. |
| Harrison, R. et al. (2006)  UK  To determine patient perceptions of joint teleconsultations (JTC), and factors contributing to satisfaction and dissatisfaction with this mode of health delivery.  Synchronous | A qualitative study using semi-structured interviews.  Virtual Outreach. Joint teleconferenced consultations with patients, their GPs and a hospital specialist  A purposive sample of n=28 participants (sourced from an RCT)  Setting= One urban and one rural area in Britain.  Scale=local  Equivocal results to patients. | Yes/ No  Thematic analysis of transcribed tape-recorded interviews.  Themes categories customer care and doctor/patient interaction. | Patients welcomed contact with their GP as they felt reassured and perceived am improved communication between the specialist and generalist. However, views were divided with some patients expressing a sense of alienation from using the technology and the impact of this on the dr-pt communication.  Using the technology was thought to offer greater convenience and punctuality of the service and lower costs.  Consultations, where a physical examination is necessary, may not be suitable for this model of telemedicine. |
| Hickson, R. et al. (2014)  USA  To systematically analyse the current literature on the state of e-consultation utilisation with a focus specifically on non-urgent primary care practice settings.  Mixed | Systematic literature review.  n=327 articles identified; n=303 excluded:  Included for review n= 24  Scale=international.  Equivocal results to patients. | No/No  Search using PubMed and Google Scholar  Study selection used in relation to the PRISMA framework.  Limited to English language only.  No details of how data was analysed | In multiple studies; females used e-consultations technology more than men.  Confusion among older patients regarding the concept of e-consultations is unjustified.  Some e-consultation implementation studies report contradictory patterns of patient use by age - younger patients making more frequent use.  Healthier patients may be more likely to utilise e-consultations and associated technologies, but there are inconsistent findings with regard to this.  Scant information about e-consultation patient-user health literacy, socio-economic indicators and differences in rural vs. urban populations. |
| Jacklin, PB. et al. (2003)  UK    To test the hypotheses that in comparison to conventional outpatient consultations joint teleconsultation (virtual outreach) would incur no increased costs to the NHS, reduce costs to patients, and reduce absences from work by patients and their carers.  Synchronous | Cost consequences study originating from a randomised control trial.  N=3170 patients identified: n=2094 eligible for inclusion and willing to participate.  N=1051 randomised to virtual outreach and n=1043 to standard outpatient appointments.  Questionnaire, data collection, index consultation.  Setting= 2 hospitals in London and Shrewsbury and n=29 GP practices in inner London and Wales.  Scale= Restricted to London, Shrewsbury and Wales.  Equivocal results to clinicians and organisations. | Yes, NHS costs, patient costs, health status (SF - 12), time spent attending index consultation, patient satisfaction.  Virtual outreach vs conventional outpatient groups.  t-tests for cost analysis between patient and NHS costs.  Sensitivity analysis | The results of this study suggest that telemedicine was not a cost-effective technology to deliver joint consultations between primary and secondary care. Findings showed that six months costs were greater for the virtual outreach group (£724 per patient) compared to the conventional outpatient group (£625): difference in means £99 ($162; €138) (95% confidence interval £10 to £187, P=0.03).  Costs of clinicians’ time to support virtual outreach were large and unlikely to be offset against subsequent savings to the NHS in the short term.  The virtual outreach group incurred savings in terms of reduced transport costs, fewer work hours lost, and less travel time.  Costs to the NHS service increased due to the initial installation of ISDN lines and video conferencing equipment. |
| Jiwa M. & Meng, X. (2013)  Australia  An exploration of GPs attitudes towards video consultations.  Asynchronous | A vignette study of GPs asked to watch 6 videos and offer a differential diagnosis and completed a survey about their views of the video consultation.  GPs were paid AUS $50 each for their time.  Settings= Of 102 invited, 47 participant GPs (47% response rate), were recruited from membership of the Curtin Health Innovation Research Network (CHIReN) a virtual network of GPs across Australia.  Scale= Regional  Equivocal results to clinicians. | Multivariable logistic regression.  Multiple regression models.  Stata version 12.1 was used to support analysis.  The theoretical framework was the Theory of Planned Behaviour | GP participants formed 3 approximately equal groups, those who would continue with video consultation, those who were ambivalent and those who would not.  GPs qualifying from an Australian University and Medical practitioners qualified for longer were equivocal about video consultations, whilst those in general practice longer and worked in group practices were more in favour.  Reticence was ascribed to the lack of opportunity to carry out a physical examination. |
| Kittler, AF. et al. (2004)  USA  To evaluate physician and non-clinical staff attitudes towards the use of e-mail system via a Patient Gateway Portal.  Asynchronous | A paper-based survey with N=24 (survey was administrated to n=43 care physicians), 56% response rate  Setting= One primary care clinic in Boston Population=113  Practice No=10; Practice size=large  Scale=local  Equivocal results to clinicians. | Descriptive analysis using counts and percentages. | Physicians felt that the Gateway impacted positively on some areas of practice including refill and referral request management and appointment scheduling.  Physicians were still, however, reluctant to adopt new information systems, especially if the systems did not directly benefit them.  Most participants remained concerned about the current lack of reimbursement for electronic communication with patients, remained unconvinced that using emails would reduce their workload, and approximately half were concerned about security. |
| Liddy C, et al (2013b)  Canada (Ontario)  A pilot study to evaluate the effectiveness and acceptability of an e- consultation system and impact for patients and health care providers.  Asynchronous | Mixed methods (interviews, focus groups and quantitative-based system utilisation) exploratory embedded design.  N=29 health care providers - (14 family physicians, 4 nurse practitioners and 11 specialists).  The pilot study ran from 1st Jan 2010 to 1st April 2011.  Scale= local  Benefit to: patients, clinicians and organisations. | No/No  Focus groups and interview data analysed using a coding framework with support from QSR NVIVO software.  Limited information on how system utilization data were analysed. | Evaluation data of uptake showed high levels of satisfaction, improvements in the integration of referrals and consultations, and avoidance of unnecessary specialist visits.  In the pilot study time, 77 consultations requests were made by 18 PCPs with a response from 11 specialists. The average response time was 5.5 days.  Less than 10% of the referrals required face-to-face follow-up.  Most nurse practitioners and physicians expressed satisfaction with the e-consultation service, many physicians commenting on its simplicity and effectiveness.  Benefits to patients included improved access to specialist care and reduced waiting times.  Primary care providers appreciated the chance to have a rapid response to clinical questions, including clarifying diagnosis, test or treatment issues. Specialist valued the interaction with their community colleagues and the opportunity to contribute to the discussion about possible referral. |
| Mehrotra, Ateev et al. (2013)  USA  To investigate the characteristics of patients who seek care via e-consultations by reviewing electronic medical records.  Asynchronous | Quantitative analysis of adult medical record data.  N=8119 (n=5165 sinusitis and n= 2954 urinary tract infections)  Identified e-consultations and office visits between January 2010 and May 2011.  Setting= Four primary care practices in the Pittsburgh, PA, region.  Scale=Regional  Equivocal results to patients and clinicians. | Yes / Comparison of patient demographics (age, gender, races, employment, marital status) between e-consultation and office visit users.  Bivariate analysis using chi-squared test.  Multivariate modelling of e-consultation use. | Convenience appears to be critical as distance and the winter season were associated with greater e-consultation use.    Female (sinusitis, 77%, n=5165; UTI, 98%, n=6954 p<0.001) and those that are employed were more likely to use an e-consultation (sinusitis, 75%, n=355; UTI, 60%, n=50, p<0.001).  People aged ≥ 65 years and over were less likely to use an e-consultation (sinusitis, 6%, n=28; UTI, 9%, n=9, p<0.001).  Analysis suggests patterns of use are related to how patients learn about e-consultations. For example, patients who see their physicians frequently are likely to have a patient portal account. This may be linked to higher trust in their physician and willingness to try other routes to care. |
| Mirsky, JB., Tieu L, Lyles c, & Sarker U. (2016)  USA (California)  To explore the use and content of patient-provider e-mails in a safety-net primary care clinic.  Asynchronous | An observational study of electronic messages comprising of content analysis of e-mail exchanges.  N= 22 patients in the sample.  Email exchange data collected from January through November 2013  Setting= General Medical Clinic, San Francisco. A Safety-net primary care clinic.  Scale= Local  Benefit to: patients | Deductive content analysis of qualitative data in email exchanges.  Descriptive statistics (frequency of medical updates, requests for action, and requests for  Information) were calculated using Excel. | Out of the 22 patients, n=15 (68%) were female, and the average age was 58 (SD=10).  No emails were related to urgent care. From all requests (n=56), the majority were for some form of action (77%, n=43, from strands). Forms of action related to medications or treatments (n=16, 29%); and a smaller number about lab tests or x-rays (n=7, 18%).  Requests for information were less common (n=19, 45% strand).  Many patient requests (n=56) were resolved (84%) in the e-mail exchanges, however, some requests resulted in 63 actions.  Email exchanges in safety-net clinics may offer a safe and efficient alternative for between visits for patients and their provider. Emails may offer increased health care access and may enhance patients’ overall engagement with their care. |
| Neville, R et al. (2004b)  UK (Scotland)  To evaluate an email service enabling communication between patients and their general practice regarding repeat prescriptions, appointment booking and clinical enquiries.  Asynchronous | Qualitative analysis of email interactions and an electronic user survey.  Population: N=150 (adult patients), N=62 (GPs);  Data collected between April 2002- December 2002  Setting= city (general practice in Dundee, Scotland, UK); Population= 7000 (patients);  Practice No= 1; Practice size= medium. Scale= single practice, hospital or clinic  Benefit to: clinicians | No/No  Quantitative analysis of frequency and time of email service.  Contents analysis of qualitative data (email contents, nature and exchanges) was based on Hahn’s classification of assistance requests | Use of an email consultation facility worked well, with patients being very satisfied with the services, and resulted in no apparent increase in GP workload.  Results suggest that there may be an unmet need amongst patients for clinical email services and that such services may have positive outcomes for patients and general practice.  The main barrier to practices setting up an email facility is likely to be attitudinal, rather than technical or logistical. |
| Nilsson M et al. (2009)  Sweden  To evaluate the feasibility and quality of uncomplicated hypertension care in rural areas using video conferencing.  The aim was to identify the proportion of subjects reaching the treatment goal at follow up.  Synchronous | A prospective non-randomised evaluation study on the use of video conferencing in the treatment of patients with hypertension.  Data Collected from March 2005 until Nov 2006.  Intervention subjects: n=91 consecutive patients with primary hypertension.  Comparison subjects: n=182 age and sex-matched patients with hypertension from a similar health centre.  Settings: n=2 surgeries with identical treatment protocols for hypertension, belonging to the same local management of a UK County Council (Lovanger Health care centre).  Scale=Local  Benefit to: patients and clinicians. | Comparison. A nurse-led surgery with doctor support vs a surgery with face to face consultations between physicians and patients.  Univariate and multivariate logistic regression was used for analysing differences between the intervention centre and the control group.  Data analysis conducted using standard software packages (SAS, SPSS v.15, Statistica v8, StatSoft Inc) | Videoconferencing was used in n=270 (91%) of the n=297 consultations in the intervention group.  Both patients and staff rated the treatment of hypertension via video conferencing equipment as feasible.  Blood pressure (systolic and diastolic) improved in both the intervention and comparison group throughout the study period.  However, a higher proportion of patients in the intervention group had reached their blood pressure treatment goals (systolic blood pressure, 140 mmHg, diastolic blood pressure, 90 mmHg) both at baseline and at follow-up, compared to the control group.  Patients treated via video conferencing had a higher probability of improving their blood pressure (OR 2.7, 95% CI 1.4–5.2). |
| North, F. et al. (2013)  USA  To assess the overall risk with messaging looking at death within 30 days of e-contact and hospitalisation & emergency department (ED) visits within 7 days following messaging.  Mixed | Retrospective analysis of secure messages and e-consultations.  Retrospective analysis of email contents between April 2010 and August 2011.  Setting: Primary Care Clinic, Rochester, Minnesota,  Messages totalled: n=7322 from n=2668 patients.  Scale: Region (Rochester Minnesota Local)  Equivocal results to patients. | Yes/ secure message or e-consultation  JMPV.9. 01 used for statistical analysis.  Fisher's exact test and Wilcoxon rank-sum test.  OR and 95% from the CI from the logistic model was used to compare differences between the secure messages and e-consultations. | The study used established telephone triage literature to guide the selection of potentially dangerous symptoms and used this information to determine whether patients were using portal messaging and e-consultations for symptoms requiring urgent evaluation.  Screening messages email subject lines for high-risk symptoms was not successful for identifying risks categories.  Six patients (n=6) were hospitalised from conditions related to a previous secure message (0.09%, n=6 of secure messages) and two (n=2) were hospitalised in relation to previous e-consultation (0. 22% of e-consultations, n=2).  Only 3.5% (n=2563) of messages were identified to have high-risk symptoms in the subject line. |
| North, F et al. (2014)  USA  To examine the potential impact of a portal messaging system in a primary care practice; including a subgroup of patients which were either high or long-term users to determine whether more portal communication would be associated with fewer face to face visits.  Mixed | A retrospective cohort study. Users of secure messaging and e-consultations. Overall patient trend patient data was used for 9009, 2010 and 2011.  Population: Mayo Clinic employees or dependents and primary care patients. N=141,000 total population/ n=2,357 primary care patients.  Setting: Mayo primary care clinic in Rochester, MN (USA) with 3 practice sites  Scale: Region (Rochester Minnesota Local)  Equivocal results to patients. | Face-to-face appointment frequencies were analysed before and after messaging using matched-pairs t-test.  Frequency data were analysed with and without adjustments for a first messaging visit. Subgroup analysis focused on high and long-term users. JMP v.9.01 software (SAS Institute) facilitate analysis. | The majority of patients sending messages were female, were white, and were employed by the Mayo Clinic/ lived locally. However, the study population was different across the Mayo and adult primary care practices in terms of gender, age and frequency of message usage.  Patients who used portal messages (secure messages, e-consultations) did not significantly change their frequency of face-to-face visits.  A nonsignificant difference was found (p=0.45) for primary care patients who sent at least one secure message or e-consultation (mean of 2.43, SD 2.3) compared to annual face-to-face visits before the first message and after (2.47, SD 2.8).  No significant visits differences were observed (mean, 2.35 annual visits per patient both before and after the first message; p = 0.93) after adjustments for first message surge. Subgroup analysis of high and long-term users found no significant change in visit frequency. |
| Padman, R et al. (2010)  USA  To evaluate eVisits in a primary care clinic, for a range of conditions, at three locations. over a three month period  Synchronous | Pilot mixed-methods evaluation study examining the adoption and use from e-consultations; including usage, survey and interview data.  N=152 e-consultations; N=11 physician satisfaction survey and N= 6 staff interviews.  August 2008 – December 2008.  Setting= 1 primary care outpatient practice associated with a major medical centre  Population=8,000, patients, all ages  Practice size=large. Scale=local.  Benefit to: patients and clinicians. | No/No  Patterns of usage and trends were analysed by counts and percentages.  Limited information about the analysis process. | Monthly e-consultation increased from 4% to 14%, 18% and 25% respectively. Females used e-consultations 3X more than men.  Out of n=152 visits logged in the study, 82% (n=125) were completed by doctors within 2 responses, suggesting e-consultations are fairly straightforward.  In general, patients found the service easy to use and were satisfied with the quality of care received.  The quality of the service was good, with fast response times and low numbers of messages exchanged before resolving an issue.  Patients were concerned about privacy and confidentiality, and some older patients found the concept confusing. Doctors had concerns about ease of use (non-intuitive & inflexible), functionality and value of the service, but acknowledged that e-consultations were increasingly important. |
| Pagliari C, et al. (2005)  UK (Scotland)  To determine the uptake of multiple eHealth facilities enabled by the NHS Scotland Electronic Clinical Communications Programme (ECCI) and to ascertain primary and secondary care users' perceptions.  Asynchronous | A retrospective questionnaire survey.  Setting: 5 Scottish regions comprising n=112 general practices and n=92 secondary care units.  Sample from across 16 Scottish Health Board regions.  Response rate to survey was 62% (n=112/182) in primary care and 37% (n=92/251) in secondary care.  Scale=regional  Benefit to: patients and clinicians. | A modified Delphi process to achieve consensus across n=37 quantitative indicators. Indicators included readiness to implement and saturation/use. | The process of implementation across Scotland was gradual. Over the observation period rates of adoption fell behind and varied across alternative facilities.  Electronic access to test results was the most frequently used facility and electronic outpatient booking the least.  Perceived benefits included convenience, ease of use, time-saving and the provision of an audit trail.  Users reported that though the system was beneficial, however, system reliability incompatibility and duplication of data hindered more widespread uptake. |
| Palen, TE. et al. (2012)  USA (Colorado)  To evaluate patient satisfaction with virtual consultations (VCs) and traditional consultations (TCs) facilitated within an electronic health record (EHRs)  Synchronous | A case-control study with an observational survey.  N=540 (VCs, 270; TCs, 270)/patient-adult/  01 June 2008 to 22 Nov 2008.  Randomly select 270 for each group from n=33390 populations  Setting=mix (group model integrated delivery system, over 500,000 members)  Practice size=large. Scale=national  Equivocal results to patients. | Analysis of descriptive statistics and two-group comparison between VCs and TCs. | More TCs than VCs requested transfer of patient care (p=0.03), assistance with diagnosis (p=0.04) or initiating treatment (p=0.04).  Within 3 weeks of the consultation request, 72.1% (n=195)of respondents reported receiving information from VCs, compared with 33.9% (n=92) of the TCs (p<0.001).  Referring physicians received information from consultants more quickly from VCs compared with TCs, but the value and application of information from both types of consultations were similar. |
| Polinski et al. (2015)  USA  To assess patients satisfaction with telehealth visits.  Synchronous | A cross sectional patient satisfaction survey  Population= 1734 (54 %) of 3303 patients completed the survey.  Data collected= January–September  2014.  Setting= Eleven clinics in California and Texas participated in the MinuteClinic telehealth pilot program  Size= regional  Benefit to: patients | No/No  Analysis conducted using the corresponding multivariate logistic regression model.  Analyses were undertaken using SAS  Enterprise Guide 5.1 (SAS Institute Inc., Cary, NC, USA). | The patient and a nurse communicate with a nurse practitioner or physician assistant (practitioner) using a two-way audio/visual monitor.  In total 70% (n=1214) of survey responders were female, and 41% had no usual place of care.  Over one third (32%, n=555) reported a preference for receiving care using telehealth. 57% (n=988) rated a telehealth visit to be just as good as a traditional in-person visit.  The multivariate model showed that patients with no medical insurance had 21% (n=364) increased odds of preferring a telehealth visit over that of a face-to-face visit (odds ratio [OR]=1.21; 95 % confidence interval [CI], 1.02–1.43).  Female patients also had 1.75 odds of liking telehealth visits. (95% CI, 1.05, –2.86).  The motivation for use included convenience and quality of care. |
| Popeski et al. (2015)  Canada  To describe the utilization and adoption of email in the clinical care of patients with diabetes;  Identifying barriers to and facilitators of adoption.  Asynchronous | A mixed-methods study (quantitative,  self-administered questionnaire and 5 focus groups)  Population= 16 diabetes care providers (9 physicians, 7 AHPs)  Data collected= focus groups held between June 2014 and September 2014.  Setting= A diabetes care and education centre in Calgary (Canada)  Size= regional  Benefit to: Care providers | No/No  Descriptive statistics were used to examine participant characterises using STATA 12 (StataCorp, College Station, Texas, USA).    Thematic analysis was conducted using NVivo  v.10.0 (QSR International, Melbourne, Australia). | Physicians used email less than AHPs. Professional groups differed in their view about whether email communication would improve the quality of health care delivery; with physicians’ perceiving email as an inconsistent resource.  Professional groups also differed in their views about the time it takes to respond to emails, with AHPs reporting emails would take less time than a voicemail but physicians viewing emails to be similar to that of a telephone call; and as such would take more time.  Five themes arose from the qualitative data including:  1) Barriers to use included concerns about increased patient access to care providers, inappropriate e-mails from patients, technical literacy and perceived workload rises.  2) Perceived benefits included improved communication between providers and patients and offering a record of that exchange.  3) Risks were largely discussed in terms of medicolegal liability.  4) Safeguards and risk mitigation emerged in terms of redirecting messages to other teams, to be acted on in a timely manner.  5) Compensation for services emerged when talking to physicians in terms of billing for email use, and the appropriate embedding of email within the workday, as an additional task – over and above the daily tasks of medical practice. |
| Ralston, JD. et al. (2009)  USA (Washington state and North Idaho)  To evaluate the characteristics of patients who use secure electronic messaging with their health care provider, in an integrated group practice.  Asynchronous | A cross-sectional cohort study.  Population - n=175,909 (eligible for study from n=300,000 group members); adults patients  January 2004 – March 20005  Setting = Primary care providers in a Group practice. Practice No= 20  Practice size= large. Scale=regional  Equivocal results for patients. | Retrospective analysis of secure messaging using logistic regression and descriptive statistics. | Patient web site messaging users were more likely to be female (OR, 1.15; 95% CI, 1.10-1.19) and have greater overall morbidity (OR, 5.64; 95%, CI, 5.07-6.28, comparing high or very high to very low overall morbidity).  Messaging users were more likely to be middle-aged (between 50-65 years) and less likely to be insured by Medicaid.  Patients less likely to use secure messaging was associated with enrollees age over 65 years (OR, 0.65; CI, 0.59-0.71) and Medicaid insurance vs. commercial insurance (OR, 0.81; 95%, CI, 0.68-0.96).  This may be due to differences in resources available. |
| Richards, H. et al. (2005)  UK (Scotland)  To explore the current use of and attitude towards eHealth of professions in primary care in remote areas of Scotland.  Mixed | Questionnaire/ survey  Population: GPs (n=154) in 82 practices and nurses (n=67)  Characteristics of practices <1000 = 97 (50%), 1000 + = 96 (50%).  Scale: regional  Benefit to: Clinicians and nurses | Outcome measurements listed as - reported experience of computer use according to five themes.  Data analysed using SPSS for Windows (v10) and Stata 7.0  Percentages and Chi-square/ Fisher's exact test. | 95% of respondents (n=210) had used either the internet or email.  Use of eHealth was lower with nurses than with GPs.  Most important barriers to use of eHealth were "Lack of suitable training" (55%, n=122); "high cost of buying telemedicine equipment" (54%, n=119); "increase in GP/nurse workload" (43%, n=95).  Aspects of experience with positive ratings were clinical usefulness = 76% (n=168), functioning of equipment 74% (n=164), ease of use of equipment = 74% (n=164).  Professionals were concerned about the impact of teleconsultation on patient privacy and on the consultation itself. |
| Riippa, I Linna M, and Ronkko I. (2015)  Finland  To assess the benefits and risks of providing electronic messaging services to patients with chronic conditions including a cost-effectiveness evaluation.  Asynchronous | A controlled before-and-after study including a cost-effectiveness evaluation of an electronic patient portal.  Population - n=876 eligible chronically ill patients. Data from n=137 used in the final analysis. Participants aged at least 18 years.  Data collected in 2012  Setting =Finnish public primary care. No details about practice number, size or scale.  Benefit to: patients | Yes,/ Intervention group received immediate access to a patient portal (which included secure messaging). The control group received standard care.  Effectiveness measures comprised of the Short-Form Health Survey, version 2 (SF-36v2), and the Patient Activation Measure (PAM13).  Data compared to standard care in a 6-month follow-up.  Data analysed using independent-sample t-tests (continuous variables) and chi-square tests (categorical variables). | There was a greater number of women in the intervention group (45/80, 56%) compared to the control group (26/57, 46%).  Patient-reported outcomes for patient activation at baseline were similar across intervention groups. Although patient activation improved in the intervention group; the effect was not statistically significant.  Cost-effectiveness outcomes were ambiguous, with costs of care changing according to the analytical model. As such costs decreased by an average of €91 in the unadjusted model but increased by €48 in the adjusted model.  There is a suggestion that patient activation increases in the short-term. Further studies are needed to assess the long-term effects of patient portal use on both health outcomes and cost of care. |
| Rohrer, James E. (2013)  USA  To assess patient secure e-mail message in primary care; focusing specifically on delays in response.  Asynchronous | A random sample of n=353 secure electronic messages from primary care patients.  Setting=Mixed (4 clinics from the Mayo Clinic patient portal).  April 2010 to August 2011  Scale=Local  Benefit to: patients | No/No  Descriptive statistics and univariate tests. Chi-square tests/ Fisher exact test | Generally, high rates of delayed response were higher at weekends (P<0 .001) Friday-Sunday).  A delay in responding to patients email was not related to clinic location or patient gender.  However, patients aged 50 years or older were more likely to receive a delayed response (25.7% delayed, P=.013).  8.5% of messages were not opened within 12 hours.  Nearly 20% (17.6%, n=) did not receive a response in 36 hours. |
| Roter, D. et al. (2008)  USA  To examine email exchanges between patients and physicians; focusing on contents, tone, dynamics and emotional and social dialogue.  Asynchronous | A qualitative study analysing a convenience sample of email contents between patients and physicians.  In-depth telephone interview of n=56 of the n=311 initial respondents.  N=8 of the n=56 agreed to supply copies of their last 5 email messages.  n=74 email messages (40 patient and n=34 physician) were provided.  May - October 2001.  Benefit to: patients and clinicians. | E-mails rated on a six-point scale.  Email content coded using the Roter Interactive Analysis System. | Physicians emails are shorter and more direct than those of patients, averaging half the number of statements (62 vs. 121: p<.02) and words (62 vs. 121; p<.02).  Patterns of email exchange seem similar to those of in-person visits and can be used by physicians in a patient-centred manner.  The patient-doctor relationship can be potentially supported by the email exchange through the provision of a medium through which patients can express worries and concerns and physicians can be patient-centred in the process. |
| Schattner P, Matthews M & Pinksier N.  (2008)  Australia (Melbourne)  To develop an agreed process for e-communication between GPs and other health care providers.  Mixed | Lessons from a feasibility study based in the Southern region of Melbourne using review of policy documents and semi-structured interviews.  Population=GPs and other healthcare providers  Semi-structured interviews with GPs and other key stakeholders (N=37) from a range of hospital and primary care networks; information technology (IT) experts and commercial vendors.  Scale: regional  Benefit to: clinicians and organisations | No/No  No data analysis detail provided. | Isolated regions have had limited success with e-connectivity between primary, secondary and tertiary health services in this region.  Although unable to develop an agreed process for e-communication between GPs and other health care providers, an agreement was achieved as to the best steps taken to develop this connectivity. This included bringing stakeholders together; linking with existing information and communication technology groups; ensuring GPs have a voice at new initiatives; encouraging comprehensive IT use at a GP level; assist remote access between different sectors (nursing homes & GPs) and developing multiple small scale projects across the region.  The timely and seamless access to essential clinical data would assist the provision of higher quality health care is agreed by medical bodies, government and consumers. |
| Sevean, P. et al. (2008)  Canada  An exploration of patient's and families' experiences with video telehealth consultations as a method of health care delivery in rural/remote communities in Northern Canada  Synchronous | A semi-structured video interview study.  A purposeful sample of n=10 patients and n=4 family members.  Setting= n=9 rural/remote communities (with a population range of 2000 - 10,000).  Scale = regional  January - March 2006  Benefit to: patients and family | Open-ended questions were used to explore patients/families experiences of telehealth and the differences between telehealth and traditional face-to-face visits.  Data were analysed using Bowlings (1997) 3 phase analysis.  Qualitative thematic content analysis | Three key themes emerged from patient responses:  1. Lessening the burden – in terms of travel, costs of travel, accommodation, lost wages, lost time and physical limitations (pain, fatigue, immobility).  2. Maximising supports – in relation to access to family, friends, local care providers and familiar home environment and ‘feeling of comfort’ in their own home.  3. Patients also identified technical and organisational issues that impacted on their experiences.  Patients were surprised that these visits seemed realistic and felt similar to face to face encounters.  Patients’ thought that these systems enabled health care workers to work more efficiently and enhanced communication.  Being able to visually see patients was also important for some. |
| Shimada, S. et al. (2013)  USA  To measure secure messaging (SM) implementation and identify facility characteristics associated with higher rates of adoption. The second objective was to explore the association of SM use and non-continuity care (i.e. urgent care (UC).  Asynchronous | Retrospective cohort study including a cross-sectional survey.  Population= n=132 VA facilities.  The number of facilities varied by hypothesis. Hypothesis 1 included facilities for which there was linkable survey data (N=132).  Hypothesis 2 excluded 8 facilities which had participated in a pre-implementation pilot programme and 1 recently opened VA centre.  24-months from July 2010 to June 2012  Scale= Regional  Benefit to: clinicians | (Hypothesis 1) Bivariate linear regression models and 2 sided t-tests.  (Hypothesis 2) segmented linear regression models.  Tested for autoregression using the Durbin-Watson test and for seasonality using the Dickey-Fuller Unit Root Test. | Hypotheses 1 proposed that greater availability of human/computer resources and organisational support would be positively associated with greater SM adoption. Hypothesis 2 proposed that the utilisation of UC would increase after adoption.  Human resources such as coordinators/ staff and volunteers to directly assist Veterans, computer resources (computers and rooms) and leadership support for co-ordinators were all associated with increased SM adoption rates.  Higher SM was associated with lower urgent care rates; early adopters of SM achieved a greater decrease in urgent care utilisation over time than late adopters. This work found an association linking SM and reduction in urgent care utilisation. |
| Torppa M et al. (2006)  Finland  To analysis the interaction in the patient-nurse-doctor teleconsultation in primary care.  Synchronous | A qualitative analysis of n=30 cases from a larger RCT of remote consultations in rural and northeast Finland.  N= 30 cases were chosen from 508 teleconsultations.  Randomly selected and video recorded.  Data collected= 2002  Scale=regional  Benefit to: clinicians and patients | Yes/ teleconsultations  Multiple case analysis was undertaken using a grounded theory approach. | The doctor was required to concentrate on several tasks in the teleconsultations, sometimes detracting his attention from the patient. In these situations, the patient sought empathic understanding from the nurse.  The nurse used non-verbal signs to indicate active listening, attention and empathy which resulted in the doctor feeling like an outsider.  Dr-Nurse-patient triad affected the primacy and privacy of the Dr-patient relationship.  This interaction raises challenges for the group interaction; to ensure successful doctor-nurse collaboration and positive patient outcomes. |
| Umefjord, G. et al. (2004)  Sweden  The exploration of general practitioners experiences in providing text-based consultations.  Asynchronous | Survey of n=21 general practitioners  March 2001 - no end date specified.  Setting= GPs providing online consultation in a Swedish ‘ask a doctor service’.  Practice No=n/a; Practice size=n/a. Scale= Other (internet based online consulting service)  Benefit to: clinicians | No/No  Multiple choices and free text questionnaire data were analysed independently by 2 authors using thematic coding processes. Numbers and percentages data provided. | GPs were stimulated and challenged by providing online consultations, despite lack of personal meeting or physical examination.  GPs were keen to improve their performance by learning more about how to undertake consultations on the internet, as this mode of contact raises a need to learn different behaviours.  Satisfaction may be related to the variety of questions and their challenging nature, so doctors did not get bored and were motivated to learn.  The lack of feedback and dialogue is a limitation of a text-based service, but may also be its asset when the enquirer wants to remain anonymous. |
| Umefjord, G. et al. (2006)  Sweden  To investigate how an 'ask the doctor' internet-based service (online asynchronous communication advice service) was used and evaluated by internet users.  Asynchronous | A national web-based survey (collecting quantitative and qualitative data).  Population=N=1223; all ages  November 2001 – January 2002  Setting= All enquirers to internet-based 'ask the doctor' service  Practice No= n/a; Practice size= n/a. Scale= Other (internet based online consulting service)  Benefit to: patients | No/No  Multiple choice and free text survey data were analysed quantitatively and qualitatively. | Almost half of the enquiries (45%, n=550) concerned a medical matter that had not been evaluated by a Dr before (n=550).  Users were able to inquire about; medical symptoms, second doctors opinion about symptoms, information on specific diseases, treatments, or medications.  After reading the answer, 43% (n=526) of the participants indicated that they would not pursue their question further having received sufficient information.  Participants appreciated the service for its convenience and flexibility, but also for the ability to reflect on the written answer without having to hurry and could read it more than once. |
| Wakefield, DS. et al. (2012)  USA  To explore differences in hypothetical interest in potential portal functions among primary care patients’ vs the interests and experiences of patients who chose to enrol and those who used the portal.  Asynchronous | Three groups of patients from one medical centre.  A survey conducted at different time points. A waiting room, paper-based survey and online enrolment & follow-up surveys,  Population=Adult patients;  Waiting Room survey n= 499/713  Enrolment survey n=79/369  Follow-up survey n=124/355  Female participants n=741.  Conducted between February 2008 – June 2009  Setting= n= 3 (family, community & internal medicine clinics; in one academic medical centre)  Practice size= not specified. Scale=not specific.  Benefit to: patients | Only data of internet users were analysed.  Analysis using descriptive statistics, Chi-square tests/ Mantel-Haenszel. | There were differences between patient that showed an interest (expectations) vs actual (enrolled) groups who reported being interested in; emailing their doctor (48%, n=79 vs 73%, n=215), prescription refill (37%, n=109 vs 52%, n=153), and viewing test results (54%, n=159 vs 75%, n=221).  There were little differences between expectations vs actual groups in terms of making appointments, viewing medication lists, or sending data to the doctor.  The portal design should be optimised with an understanding of the differences between the hypothetical and actual use. Portals and their functions must be easy to access and use, asking pts can help identify potential barriers to use. |
| Wallace, P.  (2004)  UK  To examine whether e-consultations would reduce offers of hospital follow-up appointments; reduce numbers of medical interventions/investigations, and reduce numbers of contacts with the health care systems. The examination includes whether e=consultations have a positive impact on patient satisfaction and lead to improvements in patient health status.  Synchronous | A randomised controlled trial (RCT) and economic evaluation of joint e-consultations.  Population= N=2094 patients consented to participate in this study. N=1051 virtual outreach vs N=1043 standard outpatient groups.  Setting= The Royal Free Hampstead NHS Trust, London and the Royal Shrewsbury Hospital Trust in Shropshire.  This project trained n=134 GPs from n=29 practices and n=20 consultant specialists.  Scale= national  Benefit to: patients | Yes/Yes.  Economic evaluation to compare joint e-consultations between GPs, specialists and patients vs standard outpatient appointments.  Logistic regression for binary outcomes and normal-error regression for quantitative outcomes. Also, intention-to-treat analysis. | Virtual outreach consultations result in significantly higher levels of patient satisfaction than standard outpatient appointments (mean difference 0.33 scale points [95% CI 0.23-0.43], p<0.0001), and there was a reduction in the number of tests and investigations in this group (by an average of 0.79 per patient (0.37-1.21, p=0.0002)).  However, they are variably associated with increased rates of offer of follow-up according to speciality and site.  Significant service reorganisation and provision of logistical support for arranging and conducting consultations will be required to enable virtual consultations to operate efficiently. |
| Ye, J. et al. (2010)  USA  To review systematically the role of emails in patient-provider communication in terms of email content and perspectives of providers and patients on email communication in health care.  Asynchronous | Systematic review.  N=24 studies included  Setting= primary health care/ family practice, internal medicine.  Scale=national  Inclusion criteria: empirical research; conducted in the USA and written in English and articles published from 2000 – 2008.  Exclusion criteria: that focused on providers’/patients’ use of IT (information technology) in general or other types of Internet communication such as instant messengers.  Benefit to: patients and clinicians | A systematic approach was used to produce a narrative summary. No specific detail about how data was analysed or if any framework was used. | Benefits of using email for communication with providers included convenience, increased access to the provider, improved the quality of care, and feeling more comfortable to ask questions.  Email communication between patients and providers benefits the consultation process, including the patient-provider relationship.  Training and standardised guidelines are needed to ensure that patients and providers use the email effectively.  Focus on provider's willingness to adopt email in health care and how it is used will impact on their medico-legal position (in terms vulnerability since putting information into writing would be more difficult for either party to dispute). |
| Zanaboni, P. et al. (2009)  Italy  To evaluate the use of teleconsultation services for general practitioners and patients in rural areas to obtain a second opinion for cardiac, dermatological and diabetic problems.  Synchronous | Evaluation of teleconsultations data (access, acceptance, organisational impact, effectiveness and economic). (Part of a larger study)    Data analysis between 2006 and 2008.  N=957 teleconsultation contact by N=812 patients.  Setting= Mixed (30 clinical centres servicing small rural communities).  Scale=local (Average population n=3723 per community)  Benefit to: patients, clinicians and organisations. | No detailed analysis section; however economic analysis reported to include direct costs, savings in relation to in-clinic visits, diagnostic examinations and specialist teleconsultations | The use rate of teleconsultation was 52% (48 of 94 GPs).  Patients teleconsultation users were aged between 66.9 and 18 years old, and there was no significant difference between male and female (407 and 405) users groups.  In the majority of teleconsultations (91%, 844 of 927), the specialist modified the GP's decision, a saving of NHS resources and efficiency improvement recorded in 797 cases (86%). Forty seven (n=47 cases (5%)) recorded an improvement in timeliness.  In general, most of GPs were satisfied with and trusted the teleconsultation system reporting organisational benefits for some specialisms (cardiology). The use of teleconsultations succeeded in modifying the behaviour of the GPs.  The main benefit to patients' was cost savings; particularly related to avoiding transportation costs for hospital admission (€1,000.06) or diagnostic examinations (€2700.50); impacting on the quality of life. |

**Glossary**

| Remote / virtual/  e-consultation | E-consultation via remote telecommunications, generally for the purpose of diagnosis or treatment of a patient at a site remote from the patient or primary physician. (MeSH). In this study, we only focused on direct electronic communication (e.g. email, video or SkypeTM) for conditions and symptoms between health providers and patients in a primary care setting. |
| --- | --- |
| Telemedicine | Delivery of health services via remote telecommunications. This includes interactive consultative and diagnostic services. (MeSH) |
| Teleconsultation | A general term for any consultation between doctors or between doctors and patients on a network or video link (e.g., Facetime, Internet, SkypeTM). Teleconsultation. (n.d.) Segen's Medical Dictionary [2011]. |
| Asynchronous e-consultation | In this review, we define asynchronous consultation as a consultation where an intermediary person may facilitate this contact by acting as either a triage service/ gatekeeper where emails or video contact is filtered/or an intermediate person organises the contact prior to direct contact. |
| Synchronous e- consultation | The asynchronous consultation will refer to a consultation which key participants (GP & patient) are in a separate location but are able to directly communicate via Skype, email. |
